# Harnessing publicly available genetic data to prioritize lipid modifying therapeutic targets for prevention of coronary heart disease based on dysglycemic risk

**DOI:** 10.1007/s00439-016-1647-9

**Published:** 2016-03-05

**Authors:** Vinicius Tragante, Folkert W. Asselbergs, Daniel I. Swerdlow, Tom M. Palmer, Jason H. Moore, Paul I. W. de Bakker, Brendan J. Keating, Michael V. Holmes

**Affiliations:** 1 Department of Heart and Lungs, University Medical Center Utrecht, Heidelberglaan 100, 3584CX Utrecht, The Netherlands; 2 Institute of Cardiovascular Science, University College London, 222 Euston Road, London, NW1 2DA UK; 3 Durrer Center for Cardiogenetic Research, ICIN-Netherlands Heart Institute, Utrecht, The Netherlands; 4 Department of Medicine, Imperial College London, Du Cane Road, London, W12 0NN UK; 5 Department of Mathematics and Statistics, Lancaster University, Lancaster, UK; 6 Department of Biostatistics and Epidemiology, Institute for Biomedical Informatics, University of Pennsylvania, Philadelphia, PA 19104-6021 USA; 7 Department of Medical Genetics, Center for Molecular Medicine, University Medical Center Utrecht, Utrecht, The Netherlands; 8 Department of Epidemiology, Julius Center for Health Sciences and Primary Care, University Medical Center Utrecht, Utrecht, The Netherlands; 9 Department of Surgery, Perelman School of Medicine, University of Pennsylvania, 3400 Spruce Street, Philadelphia, PA 19104 USA; 10 Center for Applied Genomics, Children’s Hospital of Philadelphia, Philadelphia, PA 19104 USA; 11 Center for Clinical Epidemiology and Biostatistics, Perelman School of Medicine, University of Pennsylvania, 3400 Spruce Street, Philadelphia, PA 19104 USA; 12 Clinical Trials Services Unit and Epidemiological Studies Unit (CTSU), Nuffield Department of Population Health, Richard Doll Building, University of Oxford, Old Road Campus, Roosevelt Drive, Oxford, OX3 7LF UK

**Keywords:** Coronary Artery Disease, Ezetimibe, Coronary Artery Disease Risk, Mendelian Randomization, Glycemic Status

## Abstract

Therapeutic interventions that lower LDL-cholesterol effectively reduce the risk of coronary artery disease (CAD). However, statins, the most widely prescribed LDL-cholesterol lowering drugs, increase diabetes risk. We used genome-wide association study (GWAS) data in the public domain to investigate the relationship of LDL-C and diabetes and identify loci encoding potential drug targets for LDL-cholesterol modification without causing dysglycemia. We obtained summary-level GWAS data for LDL-C from GLGC, glycemic traits from MAGIC, diabetes from DIAGRAM and CAD from CARDIoGRAMplusC4D consortia. Mendelian randomization analyses identified a one standard deviation (SD) increase in LDL-C caused an increased risk of CAD (odds ratio [OR] 1.63 (95 % confidence interval [CI] 1.55, 1.71), which was not influenced by removing SNPs associated with diabetes. LDL-C/CAD-associated SNPs showed consistent effect directions (binomial *P* = 6.85 × 10^−5^). Conversely, a 1-SD increase in LDL-C was causally protective of diabetes (OR 0.86; 95 % CI 0.81, 0.91), however LDL-cholesterol/diabetes-associated SNPs did not show consistent effect directions (binomial *P* = 0.15). *HMGCR*, our positive control, associated with LDL-C, CAD and a glycemic composite (derived from GWAS meta-analysis of four glycemic traits and diabetes). In contrast, *PCSK9*, *APOB*, *LPA*, *CETP*, *PLG*, *NPC1L1* and *ALDH2* were identified as “druggable” loci that alter LDL-C and risk of CAD without displaying associations with dysglycemia. In conclusion, LDL-C increases the risk of CAD and the relationship is independent of any association of LDL-C with diabetes. Loci that encode targets of emerging LDL-C lowering drugs do not associate with dysglycemia, and this provides provisional evidence that new LDL-C lowering drugs (such as PCSK9 inhibitors) may not influence risk of diabetes.

**Electronic supplementary material:**

The online version of this article (doi:10.1007/s00439-016-1647-9) contains supplementary material, which is available to authorized users.

## Introduction

LDL-cholesterol (LDL-C) is a recognized causal risk factor for coronary artery disease (CAD) (Cholesterol Treatment Trialists et al. 2012; Holmes et al. 2015). Meta-analysis of randomized clinical trials (RCTs) shows a 1 mmol/l reduction in LDL-C results in 25 % reduction in risk of CAD (Cholesterol Treatment Trialists et al. 2010). Indeed, statins remain the drug of choice to achieve LDL-C reduction, as they have proven long-term efficacy for reducing risk of cardiovascular disease and overall mortality. However, statins have been linked to increased risk of type 2 diabetes (T2D), (Preiss et al. 2011; Sattar et al. 2010) with recent evidence indicating this is mediated by an on-target effect (specifically through inhibition of 3-hydroxy-3-methylglutaryl-CoA reductase, HMGCR, the intended target of statins) (Swerdlow et al. 2015).

Whether the T2D effects of statins are specific to HMGCR inhibition or a general characteristic of LDL-C modification is of considerable importance given the ongoing development of drugs designed to reduce LDL-C. These include: (1) monoclonal antibody inhibitors of proprotein convertase subtilisin/kexin type 9 (PCSK9, encoded by the *PCSK9* gene) such as evolocumab and alirocumab (Stein et al. 2012); (2) antisense inhibitors of apolipoprotein B (apoB-100, encoded by *APOB*), such as mipomersen (Akdim et al. 2010), and; (3) the antisense inhibitor ISIS APO(a)Rx, which reduces lipoprotein(a) (Lp(a), encoded by *LPA*). These compounds are now in phase II (APO(a)Rx: NCT02160899) and phase III (mipomersen: NCT01475825; evolocumab: NCT01764633l) randomized clinical trials (RCTs) for CAD events. It is, therefore, important to characterize any glycemia-modifying properties of drugs that target protein products of *PCSK9*, *APOB* and *LPA* and to identify and prioritize additional potential therapeutic targets that alter LDL-C and risk of CAD but without causing dysglycemia.

Genetic studies provide unique opportunities to inform our understanding of disease etiology, causal mechanisms and potential therapeutic targets. Recently, data from a variety of GWAS studies have become available in the public domain, and by integrating multiple such data sets, it should become possible to obtain novel information on the potential intended and unintended consequences of drug therapy. Furthermore, these GWAS data can be exploited for Mendelian randomization analyses to generate unbiased, causal effect estimates that are free from reverse causality and confounding (Lawlor et al. 2008).

In this study, we clarify the relationship of LDL-C, CAD and dysglycemia through integrative analyses of GWAS datasets. This involves investigating: (1) whether, risk of T2D is altered as a consequence of LDL-C modification; (2) whether CAD prevention by LDL-C modification is dependent on the effect of LDL-C on diabetes; (3) whether pharmacological targets of emerging LDL-C lowering drugs associate with dysglycemia, and; (4) discovery of potential therapeutic targets for LDL-C lowering and CAD prevention that do not result in dysglycemia.

## Methods

We obtained summary-level data for: (1) LDL-C from the Global Lipids Genetics Consortium (GLGC); (2) glycemic traits from the Meta-Analyses of Glucose and Insulin-related traits Consortium (MAGIC), (3) T2D from the DIAbetes Genetics Replication And Meta-analysis (DIAGRAM) consortium, and (4) CAD from the Coronary ARtery DIsease Genome-wide Replication And Meta Analysis (CARDIoGRAM) plus The Coronary Artery Disease (C4D) Genetics, collectively known as CARDIoGRAMplusC4D consortium. The consortia provide these data openly on their respective websites: GLGC: http://www.sph.umich.edu/csg/abecasis/public/lipids2013; MAGIC: http://www.magicinvestigators.org; DIAGRAM: http://diagram-consortium.org; and, CARDIoGRAMplusC4D: http://www.cardiogramplusc4d.org. All datasets were limited to individuals of European ancestry.

We used data from GLGC as a means to harmonize estimates across the consortia. We limited our focus to SNPs that were genome-wide significant for their association with LDL-C in GLGC (at *P* < 5 × 10^−8^). We made SNPs directionally consistent across the datasets so that the effect alleles increased LDL-C. This was done by inverting alleles and corresponding beta coefficients where necessary. SNPs were mapped to the nearest loci using RefSeq (http://www.ncbi.nlm.nih.gov/refseq).

We used these data to investigate the shared association of LDL-C-related SNPs with risk of CAD, T2D and concentrations of fasting glucose. We used a nominal significance threshold of *P* < 0.05, on the basis that LDL-C is recognized as causal for CHD, and that some LDL-C loci also modify glycemic traits; thus, a Bonferroni-adjusted *P* value threshold would be too stringent in this scenario.

Mendelian randomization analysis was conducted by identifying SNPs in independent loci and *R* ^2^ < 0.8 that associated with LDL-C at *P* < 5 × 10^−8^. As a sensitivity analysis, we used a stricter *R* ^2^ threshold of <0.2. Corresponding beta coefficients or log odds (together with their standard errors) were obtained for CAD, T2D and fasting glucose and we arranged SNPs so that the estimates corresponded to the same reference allele. Using the summary estimates for each of the traits, we synthesized instrumental variable estimates for each SNP by dividing the SNP-outcome association by the SNP-LDL-C association and using the delta method to approximate the standard error (Thomas et al. 2007). This generated an instrumental variable estimate for each SNP, which we pooled using fixed-effects meta-analysis to yield a summary causal effect of the association of LDL-C with risk of CAD, T2D and concentrations of fasting glucose. To investigate the independence of effect of LDL-C on risk of CAD, we removed SNPs that associated with T2D at *P* < 0.05 and subsequently at *P* < 0.01 and repeated the analysis focusing on only the remaining SNPs. Effect estimates were compared using the three different analyses (i.e. using all SNPs, excluding SNPs at *P* < 0.01 for T2D and excluding SNPs at *P* < 0.05 for T2D).

To increase power from the published datasets, we performed a ‘glycemic burden composite’ GWAS, by meta-analyzing the SNP beta coefficients and corresponding standard errors for glycemic traits and T2D risk in MAGIC and DIAGRAM. This was conducted in METAL. The GWAS meta-analysis approach is an established technique to increase power to detect associations of SNPs with clinically related phenotypes (Ellinghaus et al. 2012; McGeachie et al. 2014; Zhernakova et al. 2011). We meta-analyzed four out of the six glycemic traits in MAGIC (fasting glucose, fasting insulin, fasting proinsulin and HbA1c) and T2D risk from DIAGRAM. We excluded HOMA-B and HOMA-IR from MAGIC based on the high correlation between the Z-scores of those traits with that of fasting insulin (Kendall’s tau rank correlation 0.95 and 0.75 for fasting insulin with HOMA-IR and HOMA-B, respectively; Supplementary Table 4), which provided little additional information for the meta-analysis results and resulted in excessive genomic inflation due to the same individuals being analyzed multiple times (including HOMA-B/HOMA-IR *λ* = 1.91; excluding HOMA-B/HOMA-IR *λ* = 1.24).

We followed two approaches to identify loci associated with differences in LDL-C and a corresponding difference in risk of CAD that were (1) related and (2) unrelated to the glycemic burden composite. First, SNP pruning was conducted by LD pruning under an *r* ^2^ < 0.5 threshold and using the “clumping” function in Plink to prioritize lead SNPs based on *P* value with the glycemic burden composite. This reduced the number of SNPs from ~2.6 M to ~440 k SNPs. SNPs that associated with LDL-C (*P* < 5 × 10^−8^), CAD (*P* < 0.05) and did not associate with the glycemic burden composite (*P* > 0.05) were taken forward. Moreover, we selected only genes that did not have an over-representation of SNPs associated with the glycemic burden (Fisher’s exact *P* > 0.05). Second, we then repeated this step looking for loci associating with LDL-C, CAD and the glycemic burden—these loci were taken forward and we selected only those that had an over-representation of SNPs that associated with the glycemic burden composite (Fisher’s exact *P* < 0.05). We calculated Fisher's exact tests with EVA (available at http://www.exploratoryvisualanalysis.org/).

To investigate whether drugs exist that target the proteins encoded by these genes, we used publicly available drug-gene interaction databases. For small molecules, we used chEMBL (Gaulton et al. 2012), a repository of experimental molecules (most of which have not been fully developed and are of unknown efficacy), developed mainly by the pharmaceutical industry. For already marketed drugs, we used an integrated database, DGIdb (http://dgidb.genome.wustl.edu), which incorporates several drug-gene interaction databases, such as DrugBank (Law et al. 2014) and PharmGKB (Thorn et al. 2010). Loci that were pharmacodynamic targets of drugs were identified through online searches (including DrugBank http://www.drugbank.ca, GeneCards http://www.genecards.org, PubMed and Google Scholar).

Finally, we investigated *PCSK9,* *APOB* and *LPA* for their association with LDL-C, CAD and glycemic burden composite. This was to investigate the likely impact on glycemic status of emerging LDL-C lowering agents at intermediate or advanced stages of clinical development. For a positive control, we examined SNPs in *HMGCR*, given their known causal effects on LDL-C, CAD and T2D (Swerdlow et al. 2015). We identified SNPs in *PCSK9*, *APOB*, *LPA* and *HMGCR* associating with LDL-C at GWAS significance in the GWAS catalog (http://www.genome.gov/gwastudies accessed October 1st, 2014) and took these forward to investigate their associations with LDL-C, CAD and glycemic burden composite in our datasets. To investigate the preponderance for SNPs in these loci to associate with the glycemic burden composite, we synthesized a Circos plot.

Analyses were conducted in R version 2.15.2, Stata version 13.1 (College Station, Texas) and METAL (http://www.sph.umich.edu/csg/abecasis/metal).

## Results

We identified SNPs from the Global Lipids Genetics Consortium [GLGC, including data from up to 95,454 individuals of European ancestry (Global Lipids Genetics et al. 2013)] that surpassed the significance threshold (*P* < 5 × 10^−8^) with LDL-C and took these forward to interrogate their relationship with CAD, T2D and fasting glucose. This resulted in 2966 SNPs associated with LDL-C, corresponding to 197 independent SNPs at 172 distinct loci.

### SNPs associated with LDL-C and CAD

**Fig. 1:**
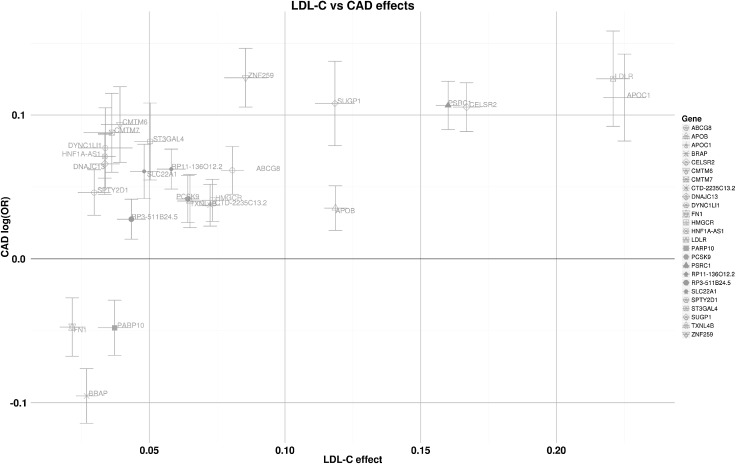
Relationship of LDL-C-associated loci with risk of CAD. The majority (22 of 25) of loci showed a consistent direction of effect with risk of CAD. LDL-C effect estimates are per SD; *whiskers* represent 95 % CI

84 of the 2966 LDL-C associated SNPs (25 of 172 loci) had nominally significant associations (at *P* < 0.05) with CAD risk in CARDIoGRAMplusC4D (including 63,746 cases and 130,681 controls of European ancestry) (Supplementary Fig. 1) (Consortium et al. 2013). Of these 25 loci, 22 (88 %) showed the same direction of effect for LDL-C and CAD (i.e., were associated both with higher LDL-C concentration and with higher risk of CAD; binomial *P* = 6.85 × 10^−5^) (Fig. 1).

**Fig. 2:**
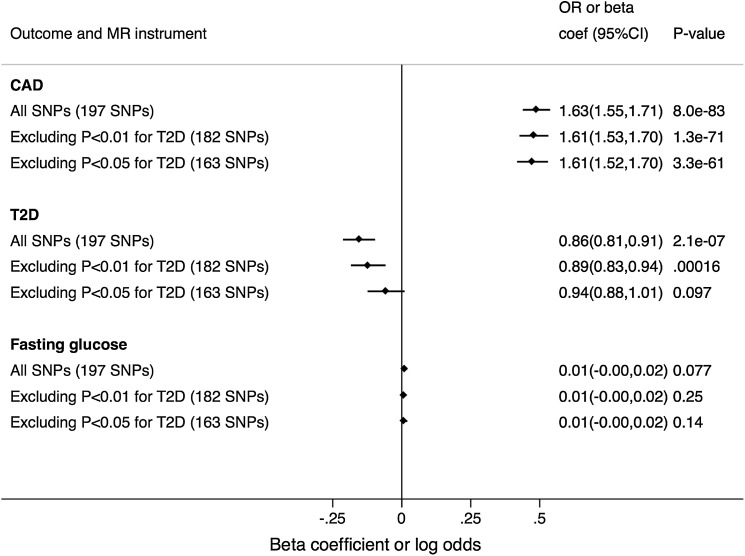
Mendelian randomization to investigate the causal relationship of a one standard deviation genetically-instrumented increase in LDL-C with risk of coronary artery disease (CAD), type 2 diabetes (T2D) and levels of fasting glucose. Single nucleotide polymorphisms (SNPs) were initially selected based on their independent association with LDL-C at *R* ^2 ^< 0.8 (*n* = 197; “All SNPs” stratum). Thereafter, we removed SNPs that associated with T2D risk at *P* < 0.01 (15 SNPs removed) and *P* < 0.05 (34 SNPs removed). Findings for the analysis using a stricter *R* ^2^ threshold (<0.2) are presented in Supplementary Figure 4

Mendelian randomization analysis of 197 independent SNPs associated with LDL-C yielded a causal OR for CAD of 1.63 (95 % confidence interval [CI] 1.55, 1.71; *P* = 8.0 × 10^−83^) per one standard deviation (SD) increase in LDL-C (Fig. 2). Using a stricter *R* ^2^ threshold (<0.2) for SNP inclusion identified 145 independent SNPs and did not materially alter the findings (Supplementary Figure 4).

### SNPs associated with LDL-C and T2D

**Fig. 3:**
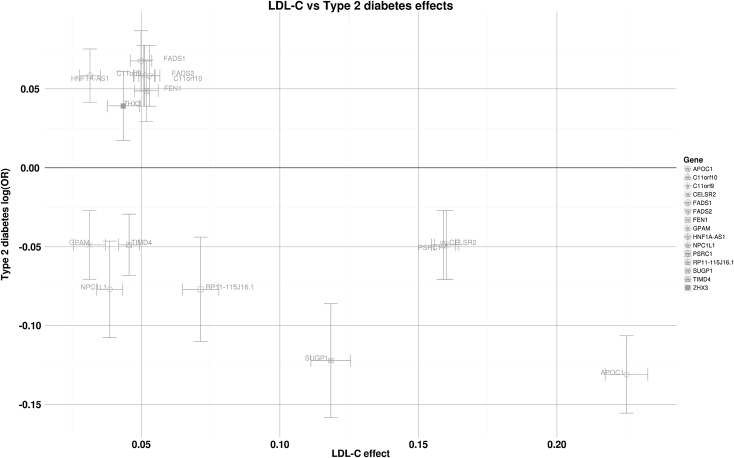
Relationship of LDL-C-associated loci with risk of T2D. Six of the 15 loci showed a positive association with T2D risk. LDL-C effect estimates are per SD; *whiskers* represent 95 % CI

61 of the 2966 LDL-C SNPs (15 of 172 loci) were nominally significant (at *P* < 0.05) for T2D in DIAGRAM (34,840 cases, 114,981 controls of European ancestry) (Supplementary Fig. 1). However, there was no clear relationship between LDL-C and T2D: of the 15 loci, 6 (40 %) showed the same direction of effect (binomial *P* = 0.15; Fig. 3).

Mendelian randomization analysis incorporating 197 independent LDL-C associated SNPs yielded a causal OR for T2D of 0.86 (95 % CI 0.81, 0.91 *P* = 2.1 × 10^−7^) per 1-SD increase in LDL-C. Removal of 15 SNPs that associated with T2D at *P* < 0.01 and 34 SNPs that associated with T2D at *P* < 0.05 resulted in a diminution of the causal OR for T2D to 0.89 (95 % CI: 0.83, 0.94; *P* = 0.002) and 0.94 (95 % CI: 0.88, 1.01; *P* = 0.10), respectively. The corresponding causal OR for CAD when the 34 SNPs associated with T2D (at *P* < 0.05) were removed remained unaltered at 1.61 (95 % CI 1.52, 1.70; *P* = 3.3 × 10^−61^) (Fig. 2). As before, using a stricter *R* ^2^ threshold (of <0.2) did not materially alter the findings (Supplementary Figure 4).

### SNPs associated with LDL-C and fasting glucose

**Fig. 4:**
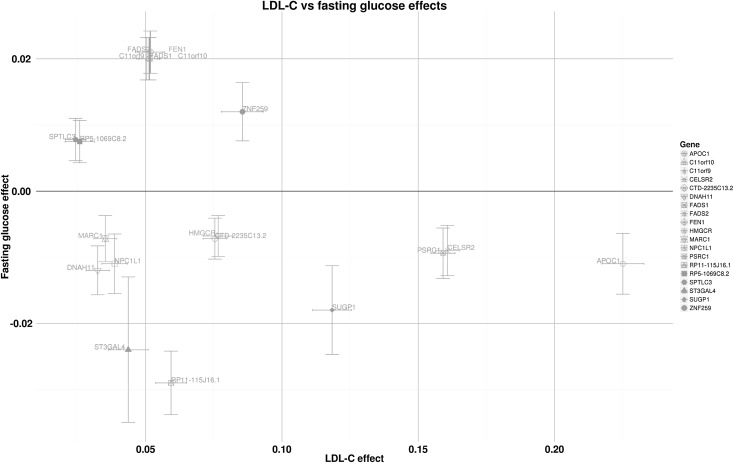
Relationship of LDL-C-associated loci with fasting glucose. Nine of 19 loci showed a positive association with fasting glucose. LDL-C effect estimates are per SD; Fasting glucose effect estimates are in mmol/l; *whiskers* represent 95 % CI

In the MAGIC consortium dataset (133,010 individuals of European ancestry), 58 SNPs (19 of 172 loci) were nominally associated (at *P* < 0.05) with fasting glucose (Supplementary Fig. 1). As with T2D, there was no clear consistency in direction of effect, with 9 of 19 loci (47 %) showing concordant directions of effect for LDL-C and fasting glucose (binomial *P* = 0.17; Fig. 4).

Mendelian randomization using 197 LDL-C-associated SNPs showed that a 1-SD increase in LDL-C had no clear effect on fasting glucose (0.009 mmol/l; 95 % CI −0.001, 0.020; *P* = 0.08).

### SNPs associated with LDL-C, CAD, fasting glucose and T2D

We next integrated data from the four traits (LDL-C, CAD, fasting glucose and T2D) using a subset of the 2966 SNPs associated with LDL-C (at *P* < 5 × 10^−8^) that also showed a nominally significant association (*P* < 0.05) with CAD risk (*n* = 84). Of these 84 SNPs, 17 were associated with fasting glucose and 13 with T2D risk. Six SNPs were associated with both T2D risk and fasting glucose. Of note, these six independent SNPs that associated with all four traits were consistent in their associations with higher LDL-C, higher CAD risk, lower fasting glucose and lower T2D risk (binomial *P* = 0.016) (Supplementary Fig. 2).

Eight of 17 SNPs associating with LDL-C, CAD and fasting glucose were located at the *HMGCR* locus. Other loci that were associated with LDL-C and CAD, and with fasting glucose and T2D included *CELSR2*, *PSRC1*, *APOC1* and *SUGP1*.

### Synthesis of a glycemic burden composite trait

To increase power to detect associations of loci with glycemic status, we developed a “glycemic burden composite”, which involved meta-analysis of associations of four glycemic traits (fasting glucose, fasting insulin, fasting proinsulin, HbA1c) together with T2D risk and included over 2.5 million SNPs in the MAGIC and DIAGRAM consortia datasets. We excluded HOMA-B and HOMA-IR from MAGIC based on the high correlation (see “Methods” and Supplementary Table 4 for more details), This identified 306 SNPs with significant associations with the glycemic burden composite (at Bonferroni-corrected *P* < 5 × 10^−8^) (Supplementary Fig. 3 and Data file S1).

We evaluated suitable loci that altered LDL-C levels and CAD risk that were free from dysglycemic effects, following three routes:

#### Loci that encode established or emerging LDL-C drug targets for CAD prevention

We focused our attention on four loci encoding targets for existing or emerging lipid-lowering agents: *HMGCR* (the intended target of the statin drugs), *PCSK9*, *APOB* and *LPA* (targets of drugs currently in phase II and phase III RCTs). We identified SNPs in the GWAS catalog (http://www.genome.gov/gwastudies, accessed October 1st 2014) in these loci that associated with LDL-C at GWAS significance.

For *HMGCR* (which served as a positive control), 5 SNPs were identified (rs12916, rs3846662, rs3846663, rs7703051, rs12654264, Table 1): all 5 *HMGCR* SNPs associated with the glycemic burden composite. The direction of effect was as expected: SNPs associated with lower LDL-C levels, lower risk of CAD and higher values for the glycemic burden composite.

**Table 1:**
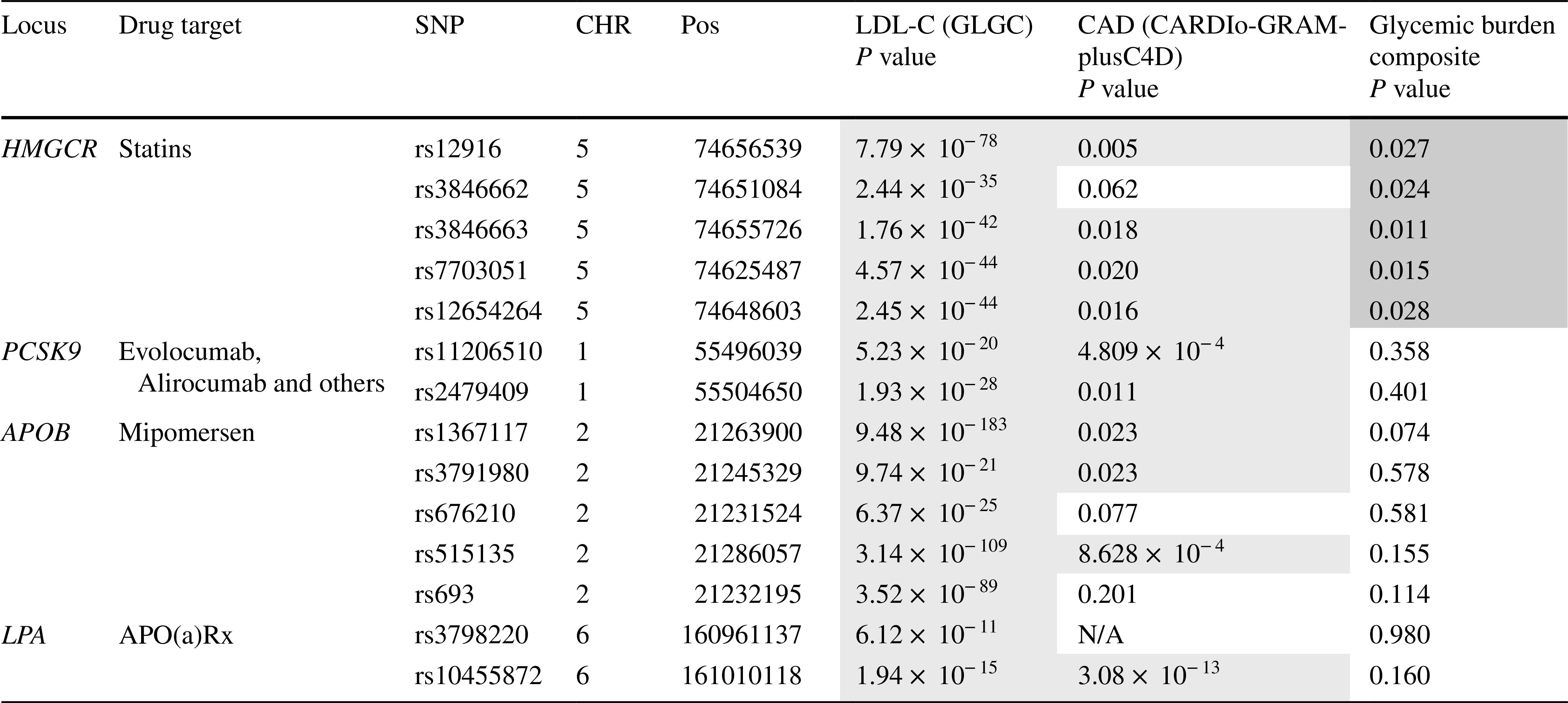
Association of variants in *HMGCR,* *PCSK9*, *APOB* and *LPA* with LDL-C, CAD risk and glycemic burden composite for GWAS Catalog SNPs (color figure online)

Blue = lower level of trait or risk of outcome; red = higher level of trait or risk of outcome

For *PCSK9*, two SNPs were identified (rs11206510, rs2479409). Both *PCSK9* SNPs associated with CAD, yet neither of them associated with the glycemic burden composite.

For *APOB*, five SNPs were identified (rs1367117, rs3791980, rs676210, rs515135, rs693), three of which associated with CAD. Again, no association of these five *APOB* SNPs was identified with the glycemic burden composite.

For *LPA*, two SNPs were identified (rs3798220, rs10455872), one of which (rs3798220) was not present in CARDIoGRAMplusC4D and no suitable proxy was available. The other SNP (rs10455872) associated with CAD. Neither of the two SNPs associated with the glycemic burden composite.

**Fig. 5:**
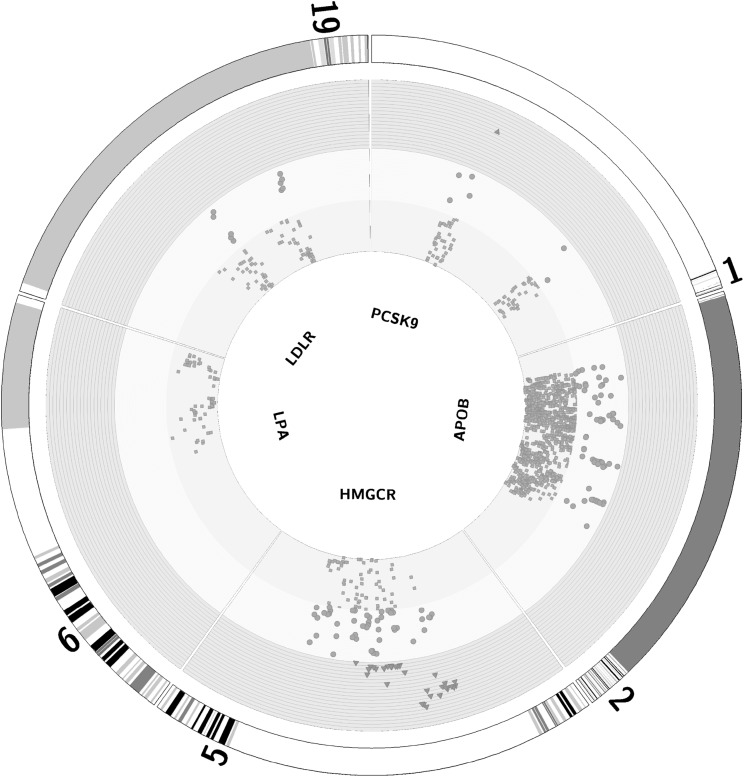
Circos diagram to show association of SNPs in *PCSK9*, *APOB*, *LPA, LDLR* and *HMGCR* with glycemic burden composite. The *outer ring* represents the genomic/chromosomal location. Each SNP is a *green*, *orange* or *red* point in the graph. *Green dots* in *green shaded ring* represent SNPs with 1 > *P* ≥ 0.05; *orange circles* in *orange shaded ring* correspond to SNPs within 0.05 > *P* ≥ 0.001 and; *red triangles* in *red shaded ring* represent SNPs with *P* < 0.001. 61 % of *HMGCR* SNPs associated with the glycemic burden composite (at *P* < 0.05) vs. less than 5 % for SNPs in *PCSK9*, *APOB* and *LPA* (color figure online)

To exploit all available data, we focused on SNPs in the same four loci (*PCSK9, APOB*, *LPA* and *HMGCR*) and evaluated the physical distribution and associations of these SNPs with the glycemic burden composite (Supplementary Fig. 3). The majority of SNPs in *HMGCR* associated with the glycemic burden composite, in contrast to the SNPs in *PCSK9*, *APOB* or *LPA* (Fig. 5).

#### Loci that associate with LDL-C and CAD, but do not associate with the glycemic burden composite

To identify potential drug targets that alter LDL-C and CAD risk with no consequence on glycemic traits, we examined SNPs in loci that associated with LDL-C (at *P* < 5 × 10^−8^) and CAD (at *P* < 0.05) but did not associate with the glycemic burden composite (*P* > 0.05). This yielded 74 loci.

Because these loci may still harbor SNPs that associate with the glycemic composite, we investigated the proportion of independent SNPs in these loci that associated with the glycemic burden composite. Loci that did not show an excess of independent SNPs associating with the glycemic burden composite (at Fisher’s exact *P* < 0.05) were investigated for druggability. In this context, “druggable” relates to a locus that encodes a protein targeted by an existing therapeutic (see “Methods” for more details).

Of the 74 loci, 62 were identified that did not harbor an excessive proportion of SNPs associating with the glycemic burden composite (Supplementary Table 1).

The protein products of 23 of the 62 loci were identified as targets of existing medications (Supplementary Table 2), see “Methods” for more details. Seven of the 23 loci were identified as pharmacodynamic targets for drugs (Table 2): *PCSK9*, *APOB*, *CETP*, *PLG, NPC1L1, LPA* and *ALDH2*. *PCSK9,* *APOB* and *LPA* have been discussed above.

**Table 2:** Loci that are pharmacodynamic targets of existing drugs identified from integrative analysis of the datasets

Gene	Drug that acts pharmacodynamically on gene protein product	Gene effect		
		LDL-C level	CAD risk	Glycemic burden composite
Drug-loci combinations with no effect on glycemic status				
*PCSK9*	ALN-PCS, BMS-PCSK9, ALN-PCS01, SPC5001	↓	↓	No effect predicted
*APOB*	SPC4955	↓	↓	No effect predicted
*CETP*	Dalcetrapib, Anacetrapib, Torcetrapib, R7232, PF-3185043, CP-800569	↓	↓	No effect predicted
*PLG*	Tenecteplase, Alteplase, Reteplase, Urokinase, Aminocaproic acid, Anistreplase, Streptokinase	↓	↓	No effect predicted
*NPC1L1*	Ezetimibe	↓	↓	No effect predicted
*ALDH2*	Disulfiram	↑	↓	No effect predicted
	Daidzin			
*LPA*	ISIS APO(a)Rx	↓	↓	No effect predicted
Drug-loci combinations that impact glycemic status				
*HMGCR*	Statins	↓	↓	↑
*SLC22A3*	Metformin	↓	↓	↓

We identified *CETP* as a druggable locus (targeted by CETP inhibitors such as anacetrapib) that has associations with LDL-C and CAD risk and an absence of association with the glycemic burden composite.

*PLG* encodes plasminogen, an enzyme that degrades plasma proteins including thrombin clots. Plasminogen is also associated with circulating lipid levels (Crutchley et al. 1989). The drugs that target the protein product of *PLG*, namely tissue plasminogen activators (e.g., streptokinase), are used clinically to degrade coronary artery thrombi in the setting of acute coronary syndrome with treatment efficacy demonstrated in RCTs (Baigent et al. 1998). Their adverse effect profile, however, includes higher risk of serious bleeding (a direct consequence of their mechanism of action), rendering them unsuitable for use in primary prevention of CAD. Furthermore, their primary mode of action is not lipid reduction, but thrombolysis.

*ALDH2* was identified to associate with LDL-C and CAD in the absence of modifying glycemic status. Interestingly, and in contrast to the other loci, *ALDH2* associated with directionally opposite effects on LDL-C and risk of CAD (Table 2). *ALDH2* encodes aldehyde dehydrogenase, responsible for metabolizing acetaldehyde, a breakdown product of alcohol. Disulfiram, a drug currently used to treat alcohol dependence, directly inhibits aldehyde dehydrogenase.

*NPC1L1* was identified to alter LDL-C and CAD risk but did not associate with the glycemic burden composite. *NPC1L1* encodes Niemann-Pick C1-Like 1 protein, a transmembrane protein that is inhibited by ezetimibe (Garcia-Calvo et al. 2005).

#### Loci that associate with LDL-C, CAD and glycemic burden composite

These loci affect glycemic status in addition to their effects on LDL-C and CAD risk, and therefore drugs modulating their encoded proteins may cause adverse dysglycemic effects. Forty independent loci were found to associate with LDL-C (*P* < 5 × 10^−8^), CAD and the glycemic burden composite (both *P* < 0.05). Of these, 11 loci were shown to have a higher than expected proportion of SNPs associated with the glycemic burden composite (Fisher’s exact *P* < 0.05), of which five loci (*HMGCR*, *SLC22A3, FADS2, ABO* and *PTPN11*) were druggable (Supplementary Table 3). Two of these loci (*HMGCR* and *SLC22A3*) have existing drugs that target them pharmacodynamically. *SLC22A3* is gaining recognition as the target of metformin, (Chen et al. 2010) a drug used to treat diabetes (by reducing blood glucose concentration), which also reduces levels of LDL-C (Keidan et al. 2002; Pentikainen et al. 1990; Robinson et al. 1998; Salpeter et al. 2008; Wulffele et al. 2004) and risk of CAD (Lamanna et al. 2011). Unlike *HMGCR*, where SNPs in the locus reduce LDL-C and CAD risk yet *increase* glycemic burden, *SLC22A3* SNPs reduce all three traits (LDL-C, CAD risk and glycemic burden).

## Discussion

We sought to clarify the relationship between LDL-C, dysglycemia and risk of CAD and shed light on potential therapeutic targets for CAD prevention that are free from dysglycemic effects. To this end, we exploited the public availability of data from several large-scale genetic consortia. Using genetic data can reliably guide which therapeutic targets should be prioritized (Holmes et al. 2013; Nelson et al. 2015).

In this study, we found that the vast majority of SNPs that influence both LDL-C and risk of CAD have the same direction of effect, that is, alleles associated with higher LDL-C levels increase CAD risk. This is further underscored by the causal effect estimate derived from Mendelian randomization, consistent with the known causal relationship between LDL-C and CAD. Both contribute further evidence in support of the so-called “LDL hypothesis”: regardless of the means, a reduction in LDL-C results in a corresponding reduction in risk of CAD (Jarcho and Keaney 2015). In contrast to the relationship of LDL-C with risk of CAD, we observed no clear patterns of association for SNPs that influence LDL-C, risk of T2D or concentrations of fasting glucose. This is despite our Mendelian randomization analysis that revealed a protective causal effect of LDL-C on the risk of T2D [directionally consistent with the relationship seen with statins and T2D risk in randomized clinical trials (Preiss et al. 2011)]. Even so, there are many loci (including druggable loci) that alter LDL-C and CAD risk that are expected to have no substantive effect on glycemic status: these include targets of novel therapies that are protein products of *PCSK9*, *APOB* and *LPA*. These findings are reinforced by the persistence of the causal relationship between LDL-C and risk of CAD even after excluding SNPs associated with T2D. Importantly, this demonstrates that the underlying causal association of LDL-C SNPs with risk of CAD remains intact, irrespective of whether SNPs also associate with T2D. Real potential therefore exists in identifying LDL-C targets that alter risk of CAD and do not impact upon glycemic status.

Of particular importance was our analysis of four candidate loci: *HMGCR*, *PCSK9*, *APOB* and *LPA*. *HMGCR* encodes 3-hydroxy-3-methyl-glutaryl-CoA reductase, the intended pharmacological target of statins, and is recognized to increase risk of T2D, both from randomized clinical trials (Preiss et al. 2011; Sattar et al. 2010) and from a recent large-scale Mendelian randomization study (Swerdlow et al. 2015). *HMGCR* SNPs that associated with LDL-C and CAD had a strong association with our glycemic burden composite. In contrast, SNPs in *APOB* and *PCSK9* that associated with LDL-C and CAD did not associate with the glycemic burden composite. These findings were reciprocated when we analyzed all available SNPs in these loci—there was a clustering of *HMGCR* SNPs associated with the glycemic burden trait that was not found for *PCSK9*, *APOB* or *LPA*. Thus, the overwhelming evidence, from several independent sources, is that drugs that target protein products of *PCSK9*, *APOB* or *LPA* should not impact upon glycemia. This is important as on-going phase III clinical trials of PCSK9 inhibitory monoclonal antibodies (e.g. evolocumab in NCT01764633; RN316 in NCT01975389 and NCT01975376; and, alirocumab in NCT01617655) and *APOB* mRNA antisense oligonucleotide inhibitor (mipomersen in NCT01475825) will most likely show beneficial effects on major clinical outcomes [as evidenced by strong genetic associations with CAD and extremely encouraging findings from large, individual (Koren et al. 2014) and pooled analysis of phase II RCTs of PCSK9 inhibition (Stein et al. 2014)]. Our findings indicate these emerging drugs are unlikely to be hampered by mechanism-based effects on glycemic status. It is therefore possible that these emerging drugs may, in future, replace statins as the drug of choice for LDL-C lowering and CAD prevention, although this is likely to follow several years of safety monitoring and patent expiration to reduce costs.

Our multi-trait meta-GWAS to quantify a glycemic burden composite enabled us to investigate potential druggable genes that alter LDL-C and CAD risk but have no appreciable effect on glycemic status. In addition to *PCSK9*, *LPA* and *APOB* loci, we identified the druggable loci *CETP*, *NPC1L1, ALDH2* and *PLG*. *CETP* is particularly interesting and controversial (Hewing and Fisher 2012; Miller 2014; Mohammadpour and Akhlaghi 2013). CETP inhibitors were developed principally to raise HDL-C with the aim of reducing CAD risk, but potent examples of these drugs also reduce LDL-C (Bloomfield et al. 2009). Phase III clinical trials of CETP inhibitors for clinical events have been conducted, the largest being dal-OUTCOMES (Schwartz et al. 2012), which randomized 15,871 patients to dalcetrapib or placebo for 31 months and was terminated early because of futility. Furthermore, meta-analyses of several phase III RCTs have failed to show cardiovascular benefit (Kaur et al. 2014; Keene et al. 2014). Of note, meta-analyses may be flawed by including torcetrapib, a CETP inhibitor that had ‘off-target’, deleterious hypertensive effects (Gutstein et al. 2012; Sofat et al. 2010) and has since been abandoned (Diener et al. 2012). Furthermore, therapeutic effects of dalcetrapib (used in dal-OUTCOMES) on LDL-C were small (Schwartz et al. 2012). Our data suggest that more potent CETP inhibitors, such as anacetrapib, that lower LDL-C (in addition to raising HDL-C) (Bloomfield et al. 2009) are likely to reduce CAD risk without any consequence on glycemic status. While ACCELERATE (NCT01687998), a phase III placebo-controlled RCT of 12,000 individuals with existing vascular disease randomized to evacetrapib has been halted for futility, (Lilly 2015) REVEAL (NCT01252953), with 30,000 individuals randomized to anacetrapib or placebo remains on-going and is anticipated to provide definitive evidence.

*NPC1L1* encodes the pharmacodynamic target of ezetimibe, an LDL-C lowering therapeutic that has, similar to CETP inhibitors, had a controversial history. Despite the effective lowering of LDL-C by ezetemibe, initial RCTs had not demonstrated its efficacy for surrogate markers of CHD or CHD events (Kastelein et al. 2008; Taylor et al. 2009). However, recent genetic studies (Myocardial Infarction Genetics Consortium I et al. 2014; Ference et al. 2015) and findings from a phase III RCT [IMPROVE-IT (Cannon et al. 2015)] provide evidence that ezetimibe is efficacious at reducing risk of CVD (McPherson and Hegele 2015). Our findings extend current knowledge to suggest that pharmacological lowering of LDL-C by ezetimibe with corresponding CAD prevention is unlikely to be accompanied by dysglycemia.

*ALDH2* is also of considerable interest. Given the recent large-scale Mendelian randomization analysis of a SNP in *ADH1B* that indicates alcohol consumption alters LDL-C and CAD risk, (Holmes et al. 2014) we have the corollary of *ALDH2*, encoding aldehyde dehydrogenase, another key enzyme in the primary metabolic pathway of alcohol. SNPs in *ALDH2* associate with an increase in LDL-C concentration and yet a reduction in CAD risk. Importantly, drugs that specifically target the protein product of *ALDH2*, used to treat alcohol dependence, such as disulfiram, should be further investigated for their effect on LDL-C and CAD risk. Preliminary studies suggest that disulfiram increases total cholesterol (Major and Goyer 1978), thus the drug may associate with a reduction in risk of CAD (in keeping with the expected pattern of association as reported in Table 2). The association of *PLG* with LDL-C and CAD is interesting: prospective studies and clinical trials show consistent associations of plasminogen with lipid levels (Crutchley et al. 1989) and CAD risk (Baigent et al. 1998; Lowe et al. 2004; Sakkinen et al. 1999). However, the mechanism-based risk of bleeding that exists with plasminogen activators renders their widespread use for CAD prevention unlikely.

Our study has several advantages. First, it demonstrates the value of exploiting data available in the public domain to conduct original analyses and answer important questions on the causal relationships between traits and diseases. In this respect, the Mendelian randomization analysis for CAD limited to SNPs not associating with T2D risk provides novel insights into disentangling the relationships between LDL-C, glycemic status and risk of CAD. Second, the now well-characterized associations of the *HMGCR* locus, selected as a positive control, with the glycemic burden composite, LDL-C and CAD were confirmed, further validating the techniques we used. The *SLC22A3* locus, of which the protein product is reported as the pharmacological target of metformin, is also noteworthy. Metformin retains a special place in the management of T2D as the only oral hypoglycemic agent that benefits both glycemic status and risk of CAD. We show that variants in *SLC22A3* alter glycemia, LDL-C and risk of CAD in a fashion that reflects the profile of actions seen with metformin in randomized trials, (Lamanna et al. 2011; Salpeter et al. 2008; Wulffele et al. 2004) providing further evidence that *SLC22A3* may well be the pharmacological target of metformin. It is intriguing that *SLC22A3* shares the same LDL-C and CAD modifying properties as *HMGCR* whereas the opposite effect on glycemia. One could speculate that metformin co-prescribed with statins could offset the diabetogenic effects of statins, whilst providing an additional means to reduce LDL-C (and CAD risk) that is independent of *HMGCR*.

Our study also has several limitations. First, use of summary-level data prevented more intricate analysis including use of covariates and conditioning, and was limited to the models used in the original analyses. However, use of summary estimates from published GWAS consortia maximizes use of all available data, thereby mitigating against publication bias, increasing power and enhancing generalizability of findings (Lin and Zeng 2010). Second, our threshold for the glycemic burden composite (*P* < 0.05) in SNPs associating with LDL-C (*P* < 5 × 10^−8^) and CAD (*P* < 0.05) may be interpreted as insufficiently stringent, given the multiple tests conducted. However, we followed up investigations of all loci for the glycemic burden composite with a Fisher’s test to identify independent loci that harbored SNPs associated with the glycemic burden composite—and in doing so, we minimized any false positives (or negatives) using this approach. The choice of a *P* value threshold of *P* < 0.05 for CAD is justifiable given the known causal association of LDL-C SNPs with CAD together with the directions of effect of SNPs on both traits, and given that all SNPs associated at GWAS significance for LDL-C. Third, gene–gene interactions may make an important contribution to the genetic architecture of disease, and such interactions may not display so-called “marginal effects”, meaning that associations arising from interactions would not be detected in conventional associated analyses (Cordell 2009; De et al. 2015). Follow-up studies could investigate the role of gene–gene interactions in this setting. Fourth, the association of *HMGCR* loci with glycemic burden composite means that, if this information was known a few decades ago, statins may not have been developed for CAD prevention. However, this is often the case as drug discovery progresses, and early drugs are superseded by drugs with a more favorable adverse effect profile, or a broader therapeutic index (Diener et al. 2012). Finally, our Mendelian randomization analyses used a “conventional” ratio approach that does not take into account potential pleiotropy of the genetic instruments. Further studies are needed to investigate whether these findings are influenced by unbalanced pleiotropy using emerging approaches such as multivariate and/or Egger-Mendelian randomization (Bowden et al. 2015; Burgess et al. 2015).

The integrative use of multiple GWAS datasets, as we report, represents a novel approach to answering critical questions on disease etiology and to inform on intended and unintended consequences of pharmacological modification of biomarkers. Real opportunities exist for academia to work together with pharmaceutical industry to translate GWAS data and maximize understanding of which therapeutic targets to prioritize based on robust, large-scale, integrative genomic analyses (Kathiresan 2015). This would facilitate discovery of safe, efficacious new therapeutics and potentially offset the exuberant costs of drug development. Indeed, drug mechanisms that have genetic support are more than twice as likely to succeed in clinical trials, (Nelson et al. 2015) and GWAS plus Mendelian randomization have been identified as key solutions to revitalizing drug development in cardiovascular disease (Fordyce et al. 2015).

In conclusion, we used publicly available data to interrogate the relationship of LDL-C-associated SNPs for their associations with CAD risk, glycemic traits and T2D risk. We identify several potential therapeutic targets that influence LDL-C and risk of CAD that do not alter glycemic status. We provide evidence that emerging drugs that target protein products of *PCSK9*, *APOB* and *LPA* are unlikely to impact upon glycemic status, and in that regard, may have advantages over statins for LDL-C lowering and prevention of CAD.

## Data and materials availability

Data retrieved from sources, as listed in Materials and Methods. Summary estimates from glycemic burden composite GWAS meta-analysis are provided in the Data file S1.

## Electronic supplementary material

Below is the link to the electronic supplementary material.

Supplementary material 1 (DOCX 1523 kb)

Supplementary material 2 (DOCX 202 kb) Data File S1: Summary estimates from GWAS meta-analysis of glycemic traits (fasting glucose, fasting insulin, fasting proinsulin and HbA1c) and type 2 diabetes to yield a glycemic burden composite
