# Supplementary material for: Harnessing publicly available genetic data to prioritize lipid modifying therapeutic targets for prevention of coronary heart disease based on dysglycemic risk
Source: Hum Genet. 2016 Mar 5;135(5):453–67. doi: 10.1007/s00439-016-1647-9 (PMC4835528; doi:10.1007/s00439-016-1647-9)
Supplement: Supplementary file 1 [file 439_2016_1647_MOESM1_ESM.docx]

**Supplementary Materials**

**Supplementary Table 1. Loci associated with LDL-C, CAD but not the glycemic burden composite.** Loci were initially filtered by those that associated with LDL-C (P<5x10^-8^), CAD (P<0.05) and did not associate with glycemic burden composite (P>0.05).

| **Locus** | **Total # SNPs in locus** | **SNPs <0.05 for glycemic burden composite** | **Proportion associated with glycemic burden composite** | **Fisher’s exact P-value** |
| --- | --- | --- | --- | --- |
| PPARG | 29 | 19 | 65.52% | <1x10^-99^ |
| HMGCR | 12 | 11 | 91.67% | <1x10^-99^ |
| DYNC2LI1 | 8 | 7 | 87.50% | <1x10^-99^ |
| SMARCA4 | 18 | 11 | 61.11% | <1x10^-99^ |
| POLK | 5 | 4 | 80% | 0.004 |
| CMTM6 | 14 | 7 | 50% | 0.006 |
| ABCG5 | 6 | 4 | 66.67% | 0.012 |
| COL4A3BP | 12 | 6 | 50% | 0.012 |
| PTPN11 | 17 | 7 | 41.18% | 0.022 |
| SLC22A3 | 33 | 11 | 33.33% | 0.025 |
| TOP1 | 35 | 11 | 31.43% | 0.039 |
| GATAD2A | 5 | 3 | 60% | 0.044 |
| SLC22A2 | 20 | 7 | 35% | 0.054 |
| LDLR | 13 | 5 | 38.46% | 0.068 |
| PSRC1 | 3 | 2 | 66.67% | 0.086 |
| HNF1A | 10 | 4 | 40% | 0.088 |
| DNM2 | 11 | 4 | 36.36% | 0.12 |
| OASL | 11 | 4 | 36.36% | 0.12 |
| BCAM | 4 | 2 | 50% | 0.151 |
| ZHX3 | 5 | 2 | 40% | 0.222 |
| NPC1L1 | 6 | 2 | 33.33% | 0.296 |
| BSND | 16 | 4 | 25% | 0.322 |
| ATXN2 | 2 | 1 | 50% | 0.328 |
| C12orf51 | 2 | 1 | 50% | 0.328 |
| TNXB | 7 | 2 | 28.57% | 0.368 |
| HAVCR1 | 12 | 3 | 25% | 0.37 |
| IGF2R | 39 | 8 | 20.51% | 0.404 |
| PLCG1 | 3 | 1 | 33.33% | 0.449 |
| SLC22A1 | 14 | 3 | 21.43% | 0.474 |
| MYBPHL | 4 | 1 | 25% | 0.548 |
| CELSR2 | 10 | 2 | 20% | 0.561 |
| PVR | 10 | 2 | 20% | 0.561 |
| RPH3A | 34 | 6 | 17.65% | 0.591 |
| PMFBP1 | 47 | 8 | 17.02% | 0.628 |
| CETP | 19 | 3 | 15.79% | 0.691 |
| PVRL2 | 10 | 1 | 10% | 0.863 |
| DHODH | 14 | 1 | 7.14% | 0.938 |
| MARCH1 | 57 | 4 | 7.02% | 0.995 |
| ABCA1 | 95 | 7 | 7.37% | 0.999 |
| APOB | 135 | 11 | 8.15% | 1 |
| SP4 | 131 | 7 | 5.34% | 1 |
| BUD13 | 182 | 12 | 6.59% | 1 |
| DHX38 | 1 | 0 | 0% | 1 |
| DMPK | 1 | 0 | 0% | 1 |
| ILF3 | 1 | 0 | 0% | 1 |
| QTRT1 | 1 | 0 | 0% | 1 |
| STK19 | 1 | 0 | 0% | 1 |
| TRAFD1 | 1 | 0 | 0% | 1 |
| ZNF180 | 1 | 0 | 0% | 1 |
| MAPKAPK5 | 8 | 0 | 0% | 1 |
| PCSK9 | 16 | 0 | 0% | 1 |
| CLPTM1 | 6 | 0 | 0% | 1 |
| SARS | 6 | 0 | 0% | 1 |
| DNAJC13 | 13 | 0 | 0% | 1 |
| KIAA1324 | 13 | 0 | 0% | 1 |
| ABCG8 | 7 | 0 | 0% | 1 |
| DCPS | 7 | 0 | 0% | 1 |
| LPA | 18 | 0 | 0% | 1 |
| C12orf43 | 4 | 0 | 0% | 1 |
| APOA5 | 5 | 0 | 0% | 1 |
| BRAP | 5 | 0 | 0% | 1 |
| NKPD1 | 5 | 0 | 0% | 1 |
| ST3GAL4 | 9 | 0 | 0% | 1 |
| OBP2B | 11 | 0 | 0% | 1 |
| ACAD10 | 2 | 0 | 0% | 1 |
| ALDH2 | 2 | 0 | 0% | 1 |
| BCL3 | 2 | 0 | 0% | 1 |
| CARM1 | 2 | 0 | 0% | 1 |
| SLC44A2 | 2 | 0 | 0% | 1 |
| TMED1 | 2 | 0 | 0% | 1 |
| ZNF259 | 2 | 0 | 0% | 1 |
| SPTY2D1 | 12 | 0 | 0% | 1 |
| TMEM116 | 12 | 0 | 0% | 1 |
| ZNF229 | 12 | 0 | 0% | 1 |

Footnote: Fisher’s exact P-value represents the test to investigate the proportion of SNPs that associate with the glycemic burden composite in each loci.

**Supplementary Table 2. Gene drug interactions among loci that alter LDL-C and risk of CAD but are free from dysglycemia, predicted by drug-gene interaction databases**

| **Gene** | **Drug:Gene interaction** | | **Pharmaco-dynamic target?** | | **Drug databases** | | | | | | | | |
| --- | --- | --- | --- | --- | --- | --- | --- | --- | --- | --- | --- | --- | --- |
|  |  |  |  |  | Clearity Found-ation Clinical Trial | | Drug-Bank | | Pharm GKB | | TEND | | TTD |
|  | | | | | | | | | | | | | |
| **Pharmacodynamic targets** | | | | | | | | | | | | | |
| *PCSK9* | ALN-PCS, BMS-PCSK9, ALN-PCS01, SPC5001 | | ✔ | | ✖ | | ✖ | | ✖ | | ✖ | | ✔ |
| *APOB* | SPC4955 | | ✔ | | ✖ | | ✖ | | ✖ | | ✖ | | ✔ |
| *NPC1L1* | Ezetimibe | ✔ | | ✖ | | ✔ | | ✖ | | ✔ | | ✔ | |
| *CETP* | Dalcetrapib, Anacetrapib, R7232, PF-3185043, Torcetrapib, CP-800569 | | ✔ | | ✖ | | ✖ | | ✖ | | ✖ | | ✔ |
| *SLC22A3* | Metformin | | ✔ | | ✖ | | ✖ | | ✔ | | ✖ | | ✖ |
| *ALDH2* | Disulfiram | | ✔ | | ✖ | | ✔ | | ✖ | | ✔ | | ✖ |
|  | Daidzin | | ✔ | | ✖ | | ✔ | | ✖ | | ✖ | | ✖ |
| *LPA* | ISIS APO(a)Rx^1^ | | ✔ | | ✖ | | ✖ | | ✖ | | ✖ | | ✖ |
|  | | | | | | | | | | | | | |
| **Other drug-gene interactions** | | | | | | | | | | | | | |
| *PTPN11* | Dodecane-trimethylamine | ✖ | | ✖ | | ✔ | | ✖ | | ✖ | | ✖ | |
| *LDLR* | Statins | ✖ | | ✖ | | ✖ | | ✔ | | ✖ | | ✖ | |
|  | Atenolol | ✖ | | ✖ | | ✖ | | ✔ | | ✖ | | ✖ | |
|  | Alpha-d-mannose, Porfimer | ✖ | | ✖ | | ✔ | | ✖ | | ✖ | | ✖ | |
| *CETP* | Pravastatin | ✖ | | ✖ | | ✖ | | ✔ | | ✖ | | ✖ | |
|  | Statins | ✖ | | ✖ | | ✖ | | ✔ | | ✖ | | ✖ | |
| *OASL* | Ribavirin, Peginterferon alfa-2b | ✖ | | ✖ | | ✖ | | ✔ | | ✖ | | ✖ | |
| *HNF1A* | Norleucine | ✖ | | ✖ | | ✔ | | ✖ | | ✖ | | ✖ | |
| *SLC22A2* | Anthracyclines | ✖ | | ✖ | | ✖ | | ✔ | | ✖ | | ✖ | |
| *APOA5* | Statins | ✖ | | ✖ | | ✖ | | ✔ | | ✖ | | ✖ | |
| *PVR* | Myristic acid, Sphingosine | ✖ | | ✖ | | ✔ | | ✖ | | ✖ | | ✖ | |
| *IGF2R* | Mecasermin, Alpha-d-mannose (-6-phosphate) | ✔ **§** | | ✖ | | ✔ | | ✖ | | ✖ | | ✖ | |
| *SLC22A1* | Tramadol, Antiemetics, antinauseants | ✖ | | ✖ | | ✖ | | ✔ | | ✖ | | ✖ | |
| *DHODH* | Leflunomide | ✔ **§§** | | ✖ | | ✔ | | ✔ | | ✔ | | ✖ | |
|  | * | ✖ | | ✖ | | ✔ | | ✖ | | ✖ | | ✖ | |
| *ABCA1* | Statins | ✖ | | ✖ | | ✖ | | ✔ | | ✖ | | ✖ | |
|  | Probucol, Adenosine triphosphate, Glyburide | ✖ | | ✖ | | ✔ | | ✖ | | ✖ | | ✖ | |
| *SARS* | L-serine | ✖ | | ✖ | | ✔ | | ✖ | | ✖ | | ✖ | |
| *C12ORF43* | Peginterferon alfa-2b, Ribavirin | ✖ | | ✖ | | ✖ | | ✔ | | ✖ | | ✖ | |
| *DCPS* | RG3039 | ✔**§§§** | | ✖ | | ✖ | | ✖ | | ✖ | | ✔ | |
|  | ** | ✖ | | ✖ | | ✔ | | ✖ | | ✖ | | ✖ | |
| *APOB* | Irbesartan | ✖ | | ✖ | | ✖ | | ✔ | | ✖ | | ✖ | |
| *ALDH2* | Guanidine, Crotonaldehyde | ✖ | | ✖ | | ✔ | | ✖ | | ✖ | | ✖ | |
| *LPA* | Aminocaproic acid | ✖ | | ✖ | | ✔ | | ✖ | | ✖ | | ✖ | |
| MAPKAPK5 | GLPG0259 | **§§§§** | | ✔ The Druggable Genome(Rask-Andersen et al. 2014) | | | | | | | | | |
| CARM1 | BMS Pyrazole Inhibitor 7F | ✖ | | ✔ (IUPHAR/BPS Guide to PHARMACOLOGY) (Pawson et al. 2014) | | | | | | | | | |
|  | Compound 17F [PMID:19632837] | ✖ | | ✔ (IUPHAR/BPS Guide to PHARMACOLOGY) (Pawson et al. 2014) | | | | | | | | | |

§ Mecamersin causes hypoglycemic; **§§** leflunomide an antitumor agent; **§§§** RG3039 in phase I RCTs for spinal muscular atrophy; §§§§ GLPG0259 clinical trial NCT01211249 for rheumatoid arthritis; ^1^*LPA* ISIS APO(a)Rx identified from clinicaltrials.gov

***** Atovaquone, (2z)-n-(3-chloro-2'-methoxybiphenyl-4-yl)-2-cyano-3-hydroxybut-2-enamide. 2-([3,5-difluoro-3'-(trifluoromethoxy)biphenyl-4-yl]amino carbonyl)cyclopent-1-ene-1-carboxylic acid, N-anthracen-2-yl-5-methyl[1,2,4]triazolo[1,5-a]pyrimidin-7-amine, Orotic acid, Undecylamine-n,n-dimethyl-n-oxide. 2-([2,3,5,6-tetrafluoro-3'-(trifluoromethoxy)biphenyl-4-yl]amino carbonyl)cyclopenta-1,3-diene-1-carboxylic acid, 2-[4-(4-chlorophenyl)cyclohexylidene]-3,4-dihydroxy-1(2h)-naphthalenone, Decylamine-n,n-dimethyl-n-oxide, Antiproliferative agent a771726, Riboflavin monophosphate, Lauryl ,dimethylamine-n-oxide, (2z)-n-biphenyl-4-yl-2-cyano-3-hydroxybut-2-enamide, 5-(aminocarbonyl)-1,1':4',1''-terphenyl-3-carboxylicacid, 5-methyl-n-[4-(trifluoromethyl)phenyl][1,2,4]triazolo[1,5-a]pyrimidin-7-amine, (2z)-2-cyano-n-(2,2'-dichlorobiphenyl-4-yl)-3-hydroxybut-2-enamide, (2z)-2-cyano-n-(3'-ethoxybiphenyl-4-yl)-3-hydroxybut-2-enamide, 3- [(3-fluoro-3'-methoxybiphenyl-4-yl)amino]carbonyl thiophene-2-carboxylic acid, Brequinar analog, 6-fluoro-2-(2'-fluoro-1,1'-biphenyl-4-yl)-3-methylquinoline-4-carboxylic acid, 3,6,9,12,15-pentaoxatricosan-1-ol, 3-([3,5-difluoro-3'-(trifluoromethoxy)biphenyl-4-yl]amino carbonyl)thiophene-2-carboxylic acid, (2z)-n-biphenyl-4-yl-2-cyano-3-cyclopropyl-3-hydroxyprop-2-enamide

****** 5-[1-(2-fluorobenzyl)piperidin-4-yl]methoxy quinazoline-2,4-diamine , mRNA cap analog n7-methyl gpppg, 5-[(1s)-1-(3-chlorophenyl)ethoxy]quinazoline-2,4-diamine, 5-[1-(2,3-dichlorobenzyl)piperidin-4-yl]methoxy quinazoline-2,4-diamine, 7n-methyl-8-hydroguanosine-5'-diphosphate, 7-methyl-gpppa, N7-methyl-guanosine-5'-monophosphate

**Supplementary Table 3. Gene drug interactions among loci that alter LDL-C, risk of CAD and glycemic burden composite**

| **Gene** | **Drug:Gene interaction** | **Pharmaco-dynamic target?** | **Gene effect** | | | | **Drug databases** | | | | |
| --- | --- | --- | --- | --- | --- | --- | --- | --- | --- | --- | --- |
|  |  |  | **LDL-C** | **CAD risk** | | **Glycemic burden composite** | ClearityFoundationClinicalTrial | Drug-Bank | Pharm-GKB | TEND | TTD |
|  | | | | | | | | | | | |
| **Pharmacodynamic targets** | | | | | | | | | | | |
| *HMGCR* | Statins | ✔ | 🡻 | 🡻 | 🡹 | | ✔ | ✔ | ✔ | ✔ | ✔ |
| *SLC22A3* | Metformin | ✔ | 🡻 | 🡻 | 🡻 | | ✖ | ✖ | ✔ | ✖ | ✖ |
|  | | | | | | | | | | | |
| **Other drug-gene interactions** | | | | | | | | | | | |
| *HMGCR* | MK-0524B | ✖ | 🡻 | 🡻 | 🡹 | | ✖ | ✖ | ✖ | ✖ | ✔ |
|  | * | ✖ |  |  |  |  | ✖ | ✔ | ✖ | ✖ | ✖ |
|  | Catecholamines | ✖ |  |  |  |  | ✖ | ✖ | ✔ | ✖ | ✖ |
| *FADS2* | Alpha-linolenic acid | ✖ | 🡻 | 🡻 | 🡻 | | ✖ | ✔ | ✖ | ✖ | ✖ |
| *ABO* | ACE inhibitors ** | ✖ | 🡻 | 🡻 | 🡻 | | ✖ | ✔ | ✖ | ✖ | ✖ |
| *PTPN1* | Stibogluconate, Dodecane-trimethylamine | ✖✖ | 🡻 | 🡻 | 🡹 | | ✖ | ✔ | ✖ | ✖ | ✖ |

***** 1,4-dithiothreitol, NADH, 2'-monophosphoadenosine 5'-diphosphoribose, Adenosine-5'-diphosphate, Co-enzyme A, 7-[3-(4-fluoro-phenyl)-1-isopropyl-1H-indol-2-YL]-3,5-dihydroxy-heptanoic acid , 3-hydroxy-3-methyl-glutaric acid

****** Octyl 3-deoxy-2-O-(6-deoxy-alpha-L-galactopyranosyl)-beta-D-xylo-hexopyranoside , Uridine-diphosphate-n-acetylgalactosamine, Beta-methyllactoside, 4-amino-2-octyloxy-6-hydroxymethyl-tetrahydro-pyran-3,5-diol, 2-hexyloxy-6-hydroxymethyl-tetrahydro-pyran-3,4,5-triol, Uridine diphosphate galactose, Octyl 3-amino-3-deoxy-2-o-(2,6-dideoxy-alpha-L-lyxo-hexopyranosyl)-beta-d-galactopyranoside, Galactose grease, 4-amino-2-hexyloxy-6-hydroxymethyl-tetrahydro-pyran-3,5-diol, 2-hexyloxy-6-hydroxymethyl-tetrahydro-pyran-3,5-diol, H type II trisaccharide, H type I trisaccharide, Uridine-5'-diphosphate

**Supplementary Table 4. Correlation matrix between traits in MAGIC and DIAGRAM.** Values represent Kendal’s tau rank correlation coefficients.

|  | MAGIC | | | | | | DIAGRAM |
| --- | --- | --- | --- | --- | --- | --- | --- |
|  | Proinsulin | HOMA-IR | HOMA-B | Fasting insulin | Hb1Ac | Fasting glucose | T2D |
| Proinsulin |  | 0.0318 | -0.0139 | 0.0211 | 0.0347 | 0.0736 | 0.0001 |
| HOMA-IR |  |  | 0.7162 | **0.9522** | 0.0339 | 0.2692 | 0.0004 |
| HOMA-B |  |  |  | **0.7547** | -0.0099 | -0.1798 | -5.51x10^-5^ |
| Fasting insulin |  |  |  |  | 0.0280 | 0.1876 | 0.0008 |
| Hb1Ac |  |  |  |  |  | 0.11216 | -0.0001 |
| Fasting glucose |  |  |  |  |  |  | -0.0003 |
| T2D |  |  |  |  |  |  |  |

The high correlation between both HOMA-IR and HOMA-B with fasting insulin meant that removal of HOMA-IR and HOMA-B from the meta-analysis (presented in Supplementary Figure 2) minimized genomic inflation (due to the same individuals being present in different analyses).

**Supplementary Figure 1.** **Venn diagram showing loci associated with LDL-C (at P<5x10^-8^) that are also nominally (P<0.05) associated with CAD risk, fasting glucose and T2D risk,**

**Supplementary Figure 2.** **Venn diagram showing SNPs associated with LDL-C (at P<5x10^-8^), and with CAD risk, fasting glucose and T2D risk (all P<0.05).** 13 SNPs associated with T2D and 17 SNPs associated with fasting glucose. The overlapping 6 SNPs all showed the same direction of effect: higher LDL-C, higher CAD risk, lower fasting glucose and lower T2D risk.

**Supplementary Figure 3.** **Glycemic burden multiple trait genome wide association analysis using 2.5 million SNPs**. Derived from combined data on four glycemic traits (fasting glucose, fasting insulin, fasting proinsulin and HbA1c) from MAGIC and T2D risk from DIAGRAM.

Chromosome

Y-axis title: P value (log-10)

**Supplementary Figure 4.** **Mendelian randomization to investigate the causality of a one standard deviation genetically-instrumented increase in LDL-C with risk of coronary artery disease (CAD), type 2 diabetes (T2D) and levels of fasting glucose.** Single nucleotide polymorphisms were initially selected based on their independent association with LDL-C (*R^2^*<0.2) (n = 145; “All SNPs” stratum). Thereafter, we removed SNPs that associated with T2D risk at P < 0.01 (11 SNPs removed) and P < 0.05 (20 SNPs removed)
