# Supplementary material for: Harnessing publicly available genetic data to prioritize lipid modifying therapeutic targets for prevention of coronary heart disease based on dysglycemic risk
Source: Hum Genet. 2016 Mar 5;135(5):453–67. doi: 10.1007/s00439-016-1647-9 (PMC4835528; doi:10.1007/s00439-016-1647-9)
Supplement: Supplementary file 2 [file 439_2016_1647_MOESM2_ESM.docx]

**Data File S1**: Summary estimates from glycemic burden composite GWAS meta analysis

| Gene | SNP | CHR | POS | A1 | A1F | Beta | SE | P value |
| --- | --- | --- | --- | --- | --- | --- | --- | --- |
| SPTA1 | rs2779116 | 1 | 158585415 | t | 0.0284 | 0.0224 | 0.004 | 2.05E-08 |
| AC074117.10 | rs7586601 | 2 | 27584666 | a | 0.4726 | -0.011 | 0.0019 | 1.38E-08 |
| NRBP1 | rs4665978 | 2 | 27648726 | t | 0.4916 | -0.0113 | 0.0019 | 4.83E-09 |
| IFT172 | rs780110 | 2 | 27685388 | a | 0.4656 | 0.0114 | 0.0019 | 3.87E-09 |
| GCKR | rs1260326 | 2 | 27730940 | t | 0.4219 | -0.018 | 0.002 | 4.48E-20 |
| GCKR | rs780094 | 2 | 27741237 | t | 0.3962 | -0.0181 | 0.002 | 2.06E-20 |
| GCKR | rs780093 | 2 | 27742603 | t | 0.3968 | -0.0177 | 0.0019 | 7.02E-20 |
| GCKR | rs1260333 | 2 | 27748624 | a | 0.4164 | -0.0145 | 0.0019 | 4.94E-14 |
| GCKR | rs2911711 | 2 | 27750546 | a | 0.4238 | 0.0148 | 0.0019 | 1.73E-14 |
| AC109829.1 | rs4665987 | 2 | 27755825 | a | 0.2538 | -0.0127 | 0.0021 | 2.40E-09 |
| AC109829.1 | rs4665991 | 2 | 27766284 | a | 0.2412 | -0.0123 | 0.0021 | 6.88E-09 |
| AC109829.1 | rs4665382 | 2 | 27783801 | t | 0.2352 | 0.0121 | 0.0021 | 1.16E-08 |
| AC109829.1 | rs10208529 | 2 | 27786188 | a | 0.2505 | 0.0121 | 0.0021 | 1.22E-08 |
| AC109829.1 | rs4665383 | 2 | 27791555 | c | 0.2324 | 0.0122 | 0.0021 | 9.22E-09 |
| C2orf16 | rs1919128 | 2 | 27801759 | a | 0.2355 | 0.012 | 0.0021 | 1.32E-08 |
| ZNF512 | rs12478841 | 2 | 27811722 | a | 0.2359 | 0.0119 | 0.0021 | 1.78E-08 |
| ZNF512 | rs6760250 | 2 | 27812252 | a | 0.246 | -0.0119 | 0.0021 | 1.64E-08 |
| ZNF512 | rs13022873 | 2 | 27815510 | a | 0.2329 | 0.0119 | 0.0021 | 1.64E-08 |
| ZNF512 | rs12467476 | 2 | 27825715 | t | 0.2502 | 0.0119 | 0.0021 | 1.76E-08 |
| ZNF512 | rs2384656 | 2 | 27832055 | a | 0.2507 | 0.012 | 0.0021 | 1.72E-08 |
| ZNF512 | rs4666002 | 2 | 27840640 | c | 0.2931 | -0.0116 | 0.0021 | 4.79E-08 |
| AC079163.1 | rs1385358 | 2 | 146369699 | t | 0.4601 | -0.0106 | 0.0019 | 4.58E-08 |
| AC079163.1 | rs7603916 | 2 | 146372665 | t | 0.4366 | 0.0107 | 0.0019 | 3.05E-08 |
| NOSTRIN | rs13029623 | 2 | 169704754 | a | 0.1831 | 0.0171 | 0.003 | 1.87E-08 |
| NOSTRIN | rs10497348 | 2 | 169718301 | a | 0.0404 | 0.03 | 0.0051 | 3.82E-09 |
| NOSTRIN | rs3931 | 2 | 169721381 | a | 0.2823 | 0.0126 | 0.0021 | 1.34E-09 |
| SPC25 | rs10497345 | 2 | 169734801 | c | 0.0255 | 0.0293 | 0.0051 | 9.95E-09 |
| SPC25 | rs11688437 | 2 | 169738539 | t | 0.3409 | 0.0134 | 0.0021 | 1.00E-10 |
| SPC25 | rs574981 | 2 | 169748422 | t | 0.3025 | 0.0161 | 0.0022 | 8.94E-14 |
| SPC25 | rs549410 | 2 | 169748843 | a | 0.2601 | 0.0149 | 0.0022 | 5.16E-12 |
| SPC25 | rs550151 | 2 | 169748860 | c | 0.3054 | 0.0157 | 0.0021 | 2.59E-13 |
| SPC25 | rs853770 | 2 | 169749841 | t | 0.277 | 0.0161 | 0.0022 | 7.00E-14 |
| SPC25 | rs477224 | 2 | 169750483 | t | 0.2887 | -0.0205 | 0.0021 | 4.77E-22 |
| SPC25 | rs512488 | 2 | 169750661 | t | 0.2973 | 0.017 | 0.0022 | 6.24E-15 |
| SPC25 | rs13431652 | 2 | 169753415 | t | 0.2909 | 0.0318 | 0.0022 | 3.71E-48 |
| SPC25 | rs12475700 | 2 | 169753676 | a | 0.3929 | 0.0207 | 0.002 | 3.67E-25 |
| SPC25 | rs1402837 | 2 | 169757354 | t | 0.2529 | 0.0293 | 0.0024 | 6.39E-35 |
| SPC25 | rs573225 | 2 | 169757541 | a | 0.343 | 0.0312 | 0.0021 | 4.26E-51 |
| SPC25 | rs2232316 | 2 | 169757562 | a | 0.0751 | 0.0204 | 0.0033 | 3.49E-10 |
| G6PC2 | rs3770568 | 2 | 169759598 | a | 0.0773 | 0.0202 | 0.0033 | 9.06E-10 |
| G6PC2 | rs16856159 | 2 | 169759903 | a | 0.0821 | 0.0197 | 0.0033 | 2.01E-09 |
| G6PC2 | rs16856161 | 2 | 169760082 | t | 0.0716 | -0.0202 | 0.0033 | 8.47E-10 |
| G6PC2 | rs560887 | 2 | 169763148 | t | 0.3338 | -0.0325 | 0.0021 | 7.72E-54 |
| G6PC2 | rs3821117 | 2 | 169763804 | t | 0.0716 | -0.0198 | 0.0033 | 1.29E-09 |
| G6PC2 | rs2232323 | 2 | 169764141 | a | 0.018 | 0.0876 | 0.013 | 1.36E-11 |
| G6PC2 | rs2232328 | 2 | 169764546 | c | 0.0731 | -0.0185 | 0.0032 | 1.30E-08 |
| SPC25 | rs17539351 | 2 | 169766560 | t | 0.1192 | -0.0405 | 0.0045 | 2.08E-19 |
| SPC25 | rs7606215 | 2 | 169766938 | a | 0.092 | -0.0198 | 0.0033 | 1.28E-09 |
| SPC25 | rs13430620 | 2 | 169768891 | a | 0.033 | 0.0638 | 0.0091 | 2.02E-12 |
| SPC25 | rs10497346 | 2 | 169771196 | t | 0.0664 | -0.0204 | 0.0033 | 8.99E-10 |
| SPC25 | rs563694 | 2 | 169774071 | a | 0.3696 | 0.0301 | 0.002 | 1.58E-51 |
| SPC25 | rs537183 | 2 | 169774646 | t | 0.3701 | 0.0308 | 0.002 | 9.82E-54 |
| ABCB11 | rs502570 | 2 | 169774959 | a | 0.4111 | -0.0307 | 0.002 | 2.96E-53 |
| ABCB11 | rs475612 | 2 | 169776746 | t | 0.3802 | -0.0303 | 0.002 | 4.57E-51 |
| ABCB11 | rs557462 | 2 | 169777595 | t | 0.3699 | 0.0307 | 0.002 | 3.77E-53 |
| ABCB11 | rs478333 | 2 | 169779156 | a | 0.4479 | 0.0193 | 0.0019 | 2.38E-23 |
| ABCB11 | rs496550 | 2 | 169779712 | t | 0.451 | -0.0189 | 0.0019 | 1.09E-22 |
| ABCB11 | rs473351 | 2 | 169779896 | t | 0.4375 | -0.0202 | 0.002 | 6.44E-24 |
| ABCB11 | rs575671 | 2 | 169780818 | a | 0.4374 | -0.0215 | 0.0019 | 8.50E-29 |
| ABCB11 | rs519887 | 2 | 169780885 | t | 0.447 | -0.0188 | 0.0019 | 1.66E-22 |
| ABCB11 | rs486981 | 2 | 169782149 | a | 0.3818 | -0.0292 | 0.002 | 2.19E-48 |
| ABCB11 | rs484066 | 2 | 169782481 | a | 0.36 | -0.0262 | 0.002 | 5.85E-40 |
| ABCB11 | rs569805 | 2 | 169782880 | a | 0.3392 | -0.0292 | 0.002 | 1.38E-48 |
| ABCB11 | rs579060 | 2 | 169783039 | t | 0.3738 | 0.0292 | 0.002 | 1.94E-48 |
| ABCB11 | rs17540154 | 2 | 169784493 | a | 0.2728 | -0.029 | 0.0025 | 1.36E-31 |
| ABCB11 | rs508506 | 2 | 169784955 | a | 0.3821 | -0.029 | 0.002 | 4.27E-47 |
| ABCB11 | rs503931 | 2 | 169785449 | a | 0.4638 | -0.0189 | 0.0019 | 2.15E-22 |
| ABCB11 | rs551754 | 2 | 169787686 | t | 0.448 | 0.0186 | 0.0019 | 4.22E-22 |
| ABCB11 | rs497692 | 2 | 169789016 | t | 0.4509 | -0.0188 | 0.0019 | 3.59E-22 |
| ABCB11 | rs494874 | 2 | 169789306 | t | 0.3763 | -0.0291 | 0.002 | 4.79E-48 |
| ABCB11 | rs16856252 | 2 | 169789720 | t | 0.0692 | -0.0195 | 0.003 | 1.09E-10 |
| ABCB11 | rs552976 | 2 | 169791438 | a | 0.3805 | -0.0289 | 0.002 | 2.65E-48 |
| ABCB11 | rs3755157 | 2 | 169792171 | t | 0.0786 | 0.0196 | 0.003 | 1.07E-10 |
| ABCB11 | rs3755158 | 2 | 169792188 | c | 0.0731 | -0.0188 | 0.003 | 5.51E-10 |
| ABCB11 | rs472614 | 2 | 169792421 | a | 0.4523 | -0.0186 | 0.0019 | 5.18E-22 |
| ABCB11 | rs565412 | 2 | 169794283 | a | 0.4479 | -0.019 | 0.0019 | 5.62E-23 |
| ABCB11 | rs567074 | 2 | 169794431 | t | 0.4398 | -0.0205 | 0.0019 | 2.06E-26 |
| ABCB11 | rs479682 | 2 | 169794687 | t | 0.4481 | -0.019 | 0.0019 | 1.18E-22 |
| ABCB11 | rs480562 | 2 | 169794771 | a | 0.4424 | 0.019 | 0.0019 | 4.70E-23 |
| ABCB11 | rs2685803 | 2 | 169796285 | a | 0.4176 | -0.019 | 0.0019 | 6.44E-23 |
| ABCB11 | rs2544367 | 2 | 169796288 | t | 0.4462 | -0.0189 | 0.0019 | 7.59E-23 |
| ABCB11 | rs2685805 | 2 | 169797060 | a | 0.4381 | -0.0189 | 0.0019 | 1.08E-22 |
| ABCB11 | rs1581397 | 2 | 169797652 | t | 0.4424 | -0.0192 | 0.0019 | 1.62E-23 |
| ABCB11 | rs2685814 | 2 | 169798619 | t | 0.4641 | -0.0194 | 0.0019 | 1.77E-23 |
| ABCB11 | rs6709087 | 2 | 169799010 | a | 0.2648 | -0.0293 | 0.0025 | 6.23E-31 |
| ABCB11 | rs853789 | 2 | 169801488 | a | 0.374 | -0.032 | 0.0021 | 3.06E-52 |
| ABCB11 | rs860510 | 2 | 169801628 | a | 0.4412 | -0.0193 | 0.0019 | 1.51E-23 |
| ABCB11 | rs853788 | 2 | 169801905 | t | 0.4426 | -0.0192 | 0.0019 | 2.09E-23 |
| ABCB11 | rs853787 | 2 | 169802252 | t | 0.3653 | 0.0304 | 0.002 | 4.17E-50 |
| ABCB11 | rs853786 | 2 | 169802310 | a | 0.443 | 0.0193 | 0.0019 | 1.33E-23 |
| ABCB11 | rs862662 | 2 | 169802329 | a | 0.4342 | 0.021 | 0.0019 | 9.88E-28 |
| ABCB11 | rs853785 | 2 | 169802594 | t | 0.4377 | 0.0193 | 0.0019 | 1.12E-23 |
| ABCB11 | rs16856261 | 2 | 169803568 | t | 0.1004 | 0.0179 | 0.0032 | 1.48E-08 |
| ABCB11 | rs853784 | 2 | 169803674 | t | 0.4482 | 0.0196 | 0.0019 | 2.92E-24 |
| ABCB11 | rs853783 | 2 | 169805511 | a | 0.4477 | -0.0196 | 0.0019 | 2.03E-24 |
| ABCB11 | rs853781 | 2 | 169806321 | a | 0.4296 | -0.0213 | 0.0019 | 2.18E-28 |
| ABCB11 | rs853780 | 2 | 169807482 | c | 0.4475 | -0.0194 | 0.0019 | 8.43E-24 |
| ABCB11 | rs1101533 | 2 | 169808522 | a | 0.4505 | -0.0195 | 0.0019 | 3.75E-24 |
| ABCB11 | rs853779 | 2 | 169809672 | t | 0.4476 | 0.0199 | 0.0019 | 1.41E-24 |
| ABCB11 | rs853778 | 2 | 169811224 | t | 0.4429 | -0.021 | 0.002 | 4.31E-27 |
| ABCB11 | rs853773 | 2 | 169814347 | a | 0.4614 | -0.024 | 0.0021 | 1.03E-30 |
| ABCB11 | rs853772 | 2 | 169814655 | t | 0.4701 | 0.0187 | 0.0019 | 4.11E-22 |
| ADCY5 | rs11708067 | 3 | 123065778 | a | 0.2244 | 0.0153 | 0.0024 | 1.58E-10 |
| ADCY5 | rs7613951 | 3 | 123070517 | t | 0.1786 | -0.014 | 0.0025 | 2.85E-08 |
| ADCY5 | rs11717195 | 3 | 123082398 | t | 0.2282 | 0.013 | 0.0024 | 3.61E-08 |
| U1 | rs7644261 | 3 | 170676134 | c | 0.2595 | 0.0122 | 0.0021 | 7.49E-09 |
| U1 | rs1905505 | 3 | 170695426 | a | 0.2615 | -0.0118 | 0.0021 | 2.30E-08 |
| U1 | rs1514895 | 3 | 170705693 | a | 0.2592 | 0.0118 | 0.0021 | 2.56E-08 |
| U1 | rs1604038 | 3 | 170709193 | t | 0.299 | -0.0117 | 0.0021 | 2.78E-08 |
| ACSL1 | rs1996546 | 4 | 185714289 | t | 0.1634 | -0.0165 | 0.0027 | 1.76E-09 |
| ACSL1 | rs735949 | 4 | 185716232 | t | 0.1739 | 0.0164 | 0.0027 | 1.66E-09 |
| ACSL1 | rs11132255 | 4 | 185718727 | a | 0.1562 | -0.0141 | 0.0026 | 4.93E-08 |
| ACSL1 | rs11936062 | 4 | 185721370 | t | 0.1562 | -0.0145 | 0.0026 | 2.16E-08 |
| HFE | rs1800562 | 6 | 26093141 | a | 0.0648 | -0.0234 | 0.0042 | 2.74E-08 |
| DGKB | rs11980500 | 7 | 14882428 | a | 0.1711 | 0.0168 | 0.0027 | 3.59E-10 |
| DGKB | rs17168486 | 7 | 14898282 | t | 0.1811 | 0.0171 | 0.0026 | 4.84E-11 |
| DGKB | rs10276674 | 7 | 14922007 | t | 0.1653 | -0.0185 | 0.0027 | 9.39E-12 |
| DGKB | rs11763864 | 7 | 14923907 | a | 0.1117 | -0.0188 | 0.0029 | 1.04E-10 |
| DGKB | rs11974348 | 7 | 15017023 | t | 0.4257 | 0.0155 | 0.0021 | 3.76E-14 |
| DGKB | rs7800343 | 7 | 15021223 | a | 0.3622 | 0.0131 | 0.0021 | 2.73E-10 |
| DGKB | rs12374901 | 7 | 15024954 | t | 0.4349 | 0.0153 | 0.002 | 2.20E-14 |
| DGKB | rs6461145 | 7 | 15026872 | a | 0.3609 | -0.013 | 0.002 | 1.27E-10 |
| DGKB | rs10950545 | 7 | 15037077 | t | 0.2788 | 0.0133 | 0.0022 | 1.69E-09 |
| DGKB | rs17168579 | 7 | 15038077 | a | 0.2748 | -0.0131 | 0.0022 | 2.60E-09 |
| DGKB | rs11971503 | 7 | 15038452 | t | 0.279 | 0.0132 | 0.0022 | 2.05E-09 |
| DGKB | rs1002061 | 7 | 15039499 | a | 0.4901 | 0.015 | 0.0019 | 1.14E-14 |
| DGKB | rs1005256 | 7 | 15039605 | a | 0.3456 | -0.0116 | 0.0021 | 1.92E-08 |
| DGKB | rs2189723 | 7 | 15040241 | t | 0.4914 | 0.0141 | 0.002 | 6.47E-13 |
| DGKB | rs2214619 | 7 | 15040698 | t | 0.3608 | 0.0116 | 0.002 | 1.25E-08 |
| DGKB | rs7811965 | 7 | 15041076 | a | 0.4911 | 0.0142 | 0.002 | 4.40E-13 |
| DGKB | rs2189725 | 7 | 15041873 | a | 0.4933 | -0.0138 | 0.002 | 1.76E-12 |
| DGKB | rs2214620 | 7 | 15042474 | a | 0.3609 | 0.0115 | 0.002 | 1.24E-08 |
| DGKB | rs10244143 | 7 | 15042959 | t | 0.4837 | -0.0143 | 0.002 | 3.08E-13 |
| DGKB | rs6461149 | 7 | 15044103 | t | 0.4364 | -0.0129 | 0.002 | 6.48E-11 |
| DGKB | rs9639218 | 7 | 15045619 | t | 0.4923 | -0.0125 | 0.0019 | 9.99E-11 |
| DGKB | rs4719430 | 7 | 15047280 | c | 0.4404 | -0.0121 | 0.0019 | 3.59E-10 |
| DGKB | rs7784091 | 7 | 15047637 | a | 0.4396 | -0.012 | 0.002 | 8.48E-10 |
| DGKB | rs6976086 | 7 | 15048611 | t | 0.4374 | 0.0118 | 0.002 | 1.33E-09 |
| DGKB | rs6976381 | 7 | 15048814 | t | 0.4489 | -0.0129 | 0.002 | 4.24E-11 |
| DGKB | rs12699673 | 7 | 15049604 | t | 0.4908 | 0.012 | 0.0019 | 4.12E-10 |
| DGKB | rs6972333 | 7 | 15051643 | a | 0.3128 | 0.0134 | 0.0023 | 7.29E-09 |
| DGKB | rs12113083 | 7 | 15051943 | c | 0.4351 | 0.0118 | 0.002 | 1.87E-09 |
| DGKB | rs6960043 | 7 | 15052860 | t | 0.4851 | -0.0119 | 0.002 | 1.29E-09 |
| DGKB | rs10267273 | 7 | 15053589 | t | 0.4663 | -0.0107 | 0.002 | 4.06E-08 |
| DGKB | rs10238161 | 7 | 15053827 | a | 0.4911 | 0.0109 | 0.002 | 3.64E-08 |
| DGKB | rs2191346 | 7 | 15053878 | c | 0.4586 | -0.0112 | 0.002 | 8.45E-09 |
| DGKB | rs2358158 | 7 | 15054063 | t | 0.4912 | 0.0108 | 0.002 | 3.52E-08 |
| DGKB | rs10264723 | 7 | 15054370 | t | 0.4821 | 0.0113 | 0.002 | 9.22E-09 |
| DGKB | rs2358159 | 7 | 15054908 | t | 0.4484 | 0.0117 | 0.002 | 4.86E-09 |
| DGKB | rs7798124 | 7 | 15055616 | a | 0.4578 | -0.0109 | 0.002 | 3.59E-08 |
| DGKB | rs7781710 | 7 | 15056045 | a | 0.4927 | -0.0109 | 0.002 | 3.38E-08 |
| DGKB | rs10950549 | 7 | 15057829 | a | 0.4932 | -0.0116 | 0.002 | 2.61E-09 |
| DGKB | rs11514706 | 7 | 15059272 | a | 0.4977 | -0.0128 | 0.002 | 1.02E-10 |
| DGKB | rs6461153 | 7 | 15059796 | c | 0.4915 | 0.0118 | 0.002 | 1.82E-09 |
| DGKB | rs4721400 | 7 | 15059906 | a | 0.4526 | -0.0115 | 0.002 | 8.86E-09 |
| AC006458.3 | rs2215383 | 7 | 15062983 | t | 0.4839 | -0.0115 | 0.0019 | 2.13E-09 |
| AC006458.3 | rs10487796 | 7 | 15063430 | a | 0.4766 | -0.0131 | 0.0019 | 1.40E-11 |
| AC006458.3 | rs10244051 | 7 | 15063833 | t | 0.4826 | -0.0129 | 0.0019 | 2.24E-11 |
| AC006458.3 | rs10258074 | 7 | 15064216 | a | 0.4835 | -0.0135 | 0.002 | 5.87E-12 |
| AC006458.3 | rs2191348 | 7 | 15064255 | t | 0.4816 | 0.013 | 0.0019 | 1.26E-11 |
| AC006458.3 | rs2191349 | 7 | 15064309 | t | 0.4668 | 0.013 | 0.0019 | 1.30E-11 |
| AC006458.3 | rs6947830 | 7 | 15064984 | a | 0.4815 | 0.013 | 0.0019 | 1.45E-11 |
| AC006458.3 | rs4719433 | 7 | 15065003 | t | 0.4828 | -0.013 | 0.0019 | 1.22E-11 |
| AC006458.3 | rs1974620 | 7 | 15065467 | t | 0.4815 | 0.0124 | 0.0019 | 1.03E-10 |
| AC006458.3 | rs1558317 | 7 | 15065564 | a | 0.464 | 0.0134 | 0.002 | 1.40E-11 |
| AC006458.3 | rs1558318 | 7 | 15065612 | a | 0.4559 | -0.0129 | 0.002 | 9.65E-11 |
| MYL7 | rs882020 | 7 | 44178743 | t | 0.1301 | 0.0162 | 0.0028 | 1.10E-08 |
| GCK | rs2268576 | 7 | 44189023 | t | 0.458 | -0.012 | 0.0021 | 9.06E-09 |
| GCK | rs2268575 | 7 | 44189274 | t | 0.1681 | -0.0247 | 0.0027 | 1.40E-19 |
| GCK | rs2268574 | 7 | 44189321 | a | 0.4598 | -0.0115 | 0.0021 | 3.59E-08 |
| GCK | rs2041547 | 7 | 44200884 | t | 0.4523 | -0.014 | 0.002 | 3.82E-12 |
| GCK | rs758989 | 7 | 44203006 | t | 0.4406 | 0.0142 | 0.0019 | 1.30E-13 |
| GCK | rs2971674 | 7 | 44203912 | a | 0.4847 | -0.0142 | 0.0019 | 1.69E-13 |
| GCK | rs2080033 | 7 | 44204322 | a | 0.4817 | 0.014 | 0.0019 | 3.15E-13 |
| GCK | rs7793213 | 7 | 44204994 | a | 0.4334 | 0.0142 | 0.0019 | 1.36E-13 |
| GCK | rs2971672 | 7 | 44205906 | a | 0.3573 | -0.0155 | 0.002 | 7.22E-15 |
| GCK | rs2908292 | 7 | 44210710 | t | 0.2068 | 0.0258 | 0.0023 | 3.74E-29 |
| GCK | rs2971671 | 7 | 44211337 | t | 0.2383 | -0.0266 | 0.0023 | 1.20E-30 |
| GCK | rs2908290 | 7 | 44216137 | a | 0.391 | 0.0158 | 0.002 | 3.34E-15 |
| GCK | rs1303722 | 7 | 44219074 | t | 0.432 | -0.0142 | 0.0019 | 2.64E-13 |
| GCK | rs2300584 | 7 | 44219338 | a | 0.234 | -0.0274 | 0.0023 | 7.96E-32 |
| GCK | rs730497 | 7 | 44223721 | a | 0.2024 | 0.0364 | 0.0026 | 2.93E-43 |
| GCK | rs2908289 | 7 | 44223942 | a | 0.2083 | 0.0365 | 0.0026 | 4.20E-45 |
| GCK | rs1799884 | 7 | 44229068 | t | 0.2033 | 0.0355 | 0.0025 | 5.92E-46 |
| GCK | rs3757840 | 7 | 44231216 | t | 0.49 | 0.0239 | 0.0021 | 3.55E-29 |
| GCK | rs2971669 | 7 | 44231778 | t | 0.2374 | 0.0342 | 0.0025 | 1.58E-41 |
| GCK | rs6975024 | 7 | 44231886 | t | 0.1994 | -0.0361 | 0.0026 | 5.56E-44 |
| GCK | rs4607517 | 7 | 44235668 | a | 0.2026 | 0.0367 | 0.0026 | 4.17E-45 |
| YKT6 | rs2971668 | 7 | 44243438 | c | 0.207 | 0.0317 | 0.0035 | 2.55E-19 |
| YKT6 | rs10278336 | 7 | 44245363 | a | 0.4397 | 0.0216 | 0.002 | 1.79E-26 |
| YKT6 | rs917793 | 7 | 44245853 | a | 0.2003 | -0.0361 | 0.0026 | 5.24E-44 |
| YKT6 | rs3824065 | 7 | 44247258 | t | 0.4443 | -0.0221 | 0.0021 | 5.72E-26 |
| YKT6 | rs2908282 | 7 | 44248828 | a | 0.2073 | 0.0363 | 0.0026 | 3.19E-45 |
| CAMK2B | rs2075076 | 7 | 44274642 | t | 0.4701 | -0.0134 | 0.0024 | 2.58E-08 |
| RP11-115J16.1 | rs983309 | 8 | 9177732 | t | 0.1052 | 0.0217 | 0.003 | 7.07E-13 |
| RP11-115J16.1 | rs6984305 | 8 | 9178268 | a | 0.1166 | 0.0215 | 0.003 | 1.20E-12 |
| RP11-115J16.1 | rs9987289 | 8 | 9183358 | a | 0.0832 | 0.0216 | 0.0033 | 5.39E-11 |
| RP11-115J16.1 | rs4841132 | 8 | 9183596 | a | 0.0834 | 0.0221 | 0.0033 | 2.37E-11 |
| RP11-115J16.1 | rs4240624 | 8 | 9184231 | a | 0.0752 | -0.0223 | 0.0033 | 1.53E-11 |
| RP11-115J16.1 | rs6601299 | 8 | 9184691 | t | 0.0798 | 0.0196 | 0.0031 | 3.29E-10 |
| RP11-115J16.1 | rs2126259 | 8 | 9185146 | t | 0.0962 | 0.0192 | 0.0032 | 1.26E-09 |
| RP11-115J16.1 | rs1461729 | 8 | 9187242 | a | 0.0992 | 0.0196 | 0.0031 | 4.52E-10 |
| RP11-115J16.1 | rs11781511 | 8 | 9192423 | a | 0.1088 | 0.0169 | 0.003 | 1.89E-08 |
| SLC30A8 | rs13266634 | 8 | 118184783 | t | 0.2435 | -0.0201 | 0.0023 | 1.65E-18 |
| SLC30A8 | rs3802177 | 8 | 118185025 | a | 0.2435 | -0.0199 | 0.0023 | 2.89E-18 |
| SLC30A8 | rs2466294 | 8 | 118185063 | c | 0.4087 | 0.0151 | 0.0022 | 3.10E-12 |
| SLC30A8 | rs11558471 | 8 | 118185733 | a | 0.252 | 0.0193 | 0.0021 | 1.45E-19 |
| SLC30A8 | rs2466292 | 8 | 118189531 | t | 0.4345 | -0.0123 | 0.0021 | 8.04E-09 |
| SLC30A8 | rs2466291 | 8 | 118190393 | a | 0.3989 | 0.0149 | 0.0023 | 3.55E-11 |
| SLC30A8 | rs2466318 | 8 | 118217307 | t | 0.431 | 0.0121 | 0.0021 | 1.80E-08 |
| SLC30A8 | rs11774700 | 8 | 118220270 | t | 0.2333 | 0.0223 | 0.0025 | 4.74E-19 |
| SLC30A8 | rs1995222 | 8 | 118229762 | t | 0.4267 | -0.0122 | 0.0021 | 3.79E-09 |
| SLC30A8 | rs951337 | 8 | 118230236 | t | 0.4269 | -0.0117 | 0.0021 | 1.36E-08 |
| SLC30A8 | rs1024068 | 8 | 118231642 | c | 0.4309 | -0.0116 | 0.0021 | 1.56E-08 |
| SLC30A8 | rs12545006 | 8 | 118232629 | a | 0.4249 | 0.0125 | 0.0021 | 1.36E-09 |
| SLC30A8 | rs7005140 | 8 | 118234147 | a | 0.4335 | 0.0117 | 0.0021 | 1.66E-08 |
| SLC30A8 | rs12545397 | 8 | 118236191 | t | 0.4548 | 0.0128 | 0.0021 | 6.39E-10 |
| SLC30A8 | rs997314 | 8 | 118236971 | t | 0.4351 | -0.013 | 0.0021 | 6.13E-10 |
| SLC30A8 | rs997313 | 8 | 118236975 | a | 0.4326 | 0.0127 | 0.0021 | 1.56E-09 |
| HK1 | rs16926246 | 10 | 71093392 | t | 0.1157 | -0.0389 | 0.0036 | 1.44E-26 |
| HK1 | rs10159477 | 10 | 71099888 | a | 0.1345 | -0.0234 | 0.0033 | 1.13E-12 |
| TCF7L2 | rs17747324 | 10 | 114752503 | t | 0.191 | -0.0191 | 0.0024 | 3.37E-15 |
| TCF7L2 | rs7901695 | 10 | 114754088 | t | 0.2807 | -0.0179 | 0.0021 | 2.37E-17 |
| TCF7L2 | rs4506565 | 10 | 114756041 | a | 0.2956 | -0.0182 | 0.0021 | 6.20E-18 |
| TCF7L2 | rs7903146 | 10 | 114758349 | t | 0.2846 | 0.0174 | 0.0021 | 7.30E-17 |
| TCF7L2 | rs10885402 | 10 | 114761697 | a | 0.4156 | -0.0106 | 0.0019 | 4.36E-08 |
| TCF7L2 | rs4132670 | 10 | 114767771 | a | 0.2969 | 0.0167 | 0.0021 | 2.05E-15 |
| TCF7L2 | rs10787472 | 10 | 114781297 | a | 0.4342 | -0.0106 | 0.0019 | 3.60E-08 |
| TCF7L2 | rs12243326 | 10 | 114788815 | t | 0.2422 | -0.0187 | 0.0022 | 2.65E-17 |
| TCF7L2 | rs7077039 | 10 | 114789077 | t | 0.436 | -0.0106 | 0.0019 | 3.94E-08 |
| TCF7L2 | rs12255372 | 10 | 114808902 | t | 0.2551 | 0.018 | 0.0022 | 1.48E-16 |
| TCF7L2 | rs11196208 | 10 | 114811316 | t | 0.4252 | -0.0105 | 0.0019 | 4.59E-08 |
| DDB2 | rs11039138 | 11 | 47249223 | a | 0.3904 | -0.0128 | 0.002 | 2.44E-10 |
| ACP2 | rs11988 | 11 | 47261260 | a | 0.3761 | -0.0121 | 0.002 | 1.60E-09 |
| NR1H3 | rs11039149 | 11 | 47276675 | a | 0.2863 | 0.0166 | 0.0022 | 2.77E-14 |
| MADD | rs10501320 | 11 | 47293799 | c | 0.2989 | -0.0164 | 0.0022 | 5.40E-14 |
| MADD | rs11606683 | 11 | 47302733 | t | 0.3884 | -0.0122 | 0.002 | 7.86E-10 |
| MADD | rs11039165 | 11 | 47312689 | a | 0.2956 | 0.0163 | 0.0022 | 5.85E-14 |
| MADD | rs749067 | 11 | 47318157 | t | 0.3736 | 0.0123 | 0.002 | 6.87E-10 |
| MADD | rs7944584 | 11 | 47336320 | a | 0.2863 | 0.0165 | 0.0022 | 2.71E-14 |
| MADD | rs11039182 | 11 | 47346723 | t | 0.306 | 0.0167 | 0.0022 | 1.93E-14 |
| MADD | rs11039183 | 11 | 47346940 | a | 0.3624 | -0.0135 | 0.002 | 2.05E-11 |
| MYBPC3 | rs11570115 | 11 | 47354905 | t | 0.1142 | 0.0181 | 0.0033 | 4.73E-08 |
| MYBPC3 | rs2697923 | 11 | 47363215 | t | 0.3662 | 0.0121 | 0.002 | 1.63E-09 |
| MYBPC3 | rs10838696 | 11 | 47363285 | a | 0.3687 | -0.0121 | 0.002 | 1.59E-09 |
| SPI1 | rs3824869 | 11 | 47378245 | t | 0.388 | -0.0187 | 0.0028 | 2.70E-11 |
| RAPSN | rs7103835 | 11 | 47461693 | a | 0.3609 | -0.0115 | 0.0021 | 4.80E-08 |
| RAPSN | rs11039244 | 11 | 47466442 | a | 0.3765 | -0.0114 | 0.002 | 2.43E-08 |
| RAPSN | rs12577643 | 11 | 47467172 | a | 0.2689 | 0.0117 | 0.0021 | 1.28E-08 |
| CELF1 | rs7933019 | 11 | 47509137 | c | 0.3497 | -0.0117 | 0.0021 | 1.71E-08 |
| CELF1 | rs6485758 | 11 | 47530024 | a | 0.3467 | -0.0118 | 0.0021 | 1.33E-08 |
| CELF1 | rs11039266 | 11 | 47532395 | t | 0.2781 | 0.012 | 0.0022 | 3.01E-08 |
| CELF1 | rs10838726 | 11 | 47568344 | c | 0.3407 | 0.0113 | 0.0021 | 4.45E-08 |
| CELF1 | rs11039290 | 11 | 47572279 | a | 0.3498 | -0.0113 | 0.0021 | 3.43E-08 |
| MTCH2 | rs7118178 | 11 | 47659135 | a | 0.284 | -0.0118 | 0.0022 | 4.19E-08 |
| NUP160 | rs7114011 | 11 | 47811309 | a | 0.295 | 0.012 | 0.0021 | 1.71E-08 |
| NUP160 | rs7131262 | 11 | 47836302 | a | 0.3355 | -0.012 | 0.0021 | 1.30E-08 |
| NUP160 | rs6485795 | 11 | 47929846 | a | 0.2889 | -0.0123 | 0.0021 | 6.93E-09 |
| RP11-676F20.1 | rs7102746 | 11 | 92666563 | t | 0.4975 | -0.0123 | 0.0022 | 2.29E-08 |
| RP11-676F20.1 | rs12222793 | 11 | 92667047 | a | 0.442 | -0.0175 | 0.0023 | 1.37E-14 |
| RP11-676F20.1 | rs4543939 | 11 | 92667658 | a | 0.375 | -0.0184 | 0.0024 | 1.48E-14 |
| RP11-676F20.1 | rs11020107 | 11 | 92667730 | c | 0.3442 | 0.0262 | 0.0021 | 2.92E-36 |
| RP11-676F20.1 | rs3847554 | 11 | 92668826 | t | 0.4071 | 0.0258 | 0.002 | 2.12E-39 |
| RP11-676F20.1 | rs12792753 | 11 | 92668975 | t | 0.3545 | -0.0295 | 0.002 | 4.58E-47 |
| RP11-676F20.1 | rs7112766 | 11 | 92672021 | t | 0.2772 | 0.0312 | 0.0022 | 1.39E-45 |
| RP11-676F20.1 | rs1387153 | 11 | 92673828 | t | 0.2756 | 0.0342 | 0.0022 | 1.10E-53 |
| RP11-676F20.1 | rs11523890 | 11 | 92679778 | t | 0.3432 | 0.0302 | 0.002 | 4.39E-50 |
| RP11-676F20.1 | rs10830956 | 11 | 92681013 | t | 0.2866 | 0.0335 | 0.0022 | 6.22E-52 |
| RP11-676F20.1 | rs10765573 | 11 | 92683332 | a | 0.3288 | 0.0302 | 0.0021 | 4.35E-49 |
| RP11-676F20.1 | rs7933855 | 11 | 92684322 | a | 0.3756 | 0.0271 | 0.002 | 1.21E-42 |
| RP11-676F20.1 | rs7936247 | 11 | 92690032 | t | 0.3103 | 0.0301 | 0.0021 | 1.92E-48 |
| RP11-676F20.1 | rs11020124 | 11 | 92690661 | t | 0.2867 | -0.0343 | 0.0023 | 1.81E-50 |
| RP11-676F20.1 | rs2166706 | 11 | 92691532 | t | 0.3901 | -0.0274 | 0.002 | 4.39E-43 |
| RP11-676F20.1 | rs10830961 | 11 | 92694757 | a | 0.3908 | -0.0285 | 0.002 | 2.46E-45 |
| RP11-676F20.1 | rs10830962 | 11 | 92698427 | c | 0.3912 | -0.0273 | 0.002 | 6.39E-42 |
| MTNR1B | rs4753426 | 11 | 92701596 | t | 0.4567 | -0.021 | 0.0021 | 4.79E-24 |
| MTNR1B | rs7951037 | 11 | 92706175 | a | 0.4633 | 0.0214 | 0.002 | 2.24E-26 |
| MTNR1B | rs10765576 | 11 | 92706944 | a | 0.4834 | -0.0212 | 0.002 | 6.71E-26 |
| MTNR1B | rs10830963 | 11 | 92708710 | c | 0.2992 | -0.0382 | 0.0023 | 2.86E-61 |
| MTNR1B | rs11020131 | 11 | 92713376 | a | 0.042 | 0.0629 | 0.0086 | 2.89E-13 |
| MTNR1B | rs3781638 | 11 | 92713507 | t | 0.4655 | 0.0215 | 0.002 | 2.15E-27 |
| MTNR1B | rs1562444 | 11 | 92715849 | a | 0.491 | 0.0267 | 0.0026 | 3.64E-24 |
| MTNR1B | rs4753072 | 11 | 92717405 | a | 0.4913 | 0.022 | 0.0019 | 2.43E-30 |
| MTNR1B | rs4753073 | 11 | 92717475 | a | 0.4955 | 0.0218 | 0.0019 | 9.15E-30 |
| MTNR1B | rs1447350 | 11 | 92718127 | c | 0.4993 | 0.0218 | 0.0019 | 7.62E-30 |
| MTNR1B | rs1447351 | 11 | 92718163 | a | 0.4968 | -0.0218 | 0.0019 | 1.10E-29 |
| MTNR1B | rs4611171 | 11 | 92719207 | t | 0.4969 | -0.0207 | 0.0019 | 4.68E-27 |
| MTNR1B | rs1597023 | 11 | 92721319 | a | 0.495 | 0.0211 | 0.0019 | 7.35E-28 |
| MTNR1B | rs4406791 | 11 | 92721787 | a | 0.4932 | -0.0213 | 0.0019 | 1.33E-28 |
| MTNR1B | rs1447352 | 11 | 92722761 | a | 0.4952 | 0.0212 | 0.0019 | 3.55E-28 |
| MTNR1B | rs7121092 | 11 | 92724351 | t | 0.496 | -0.058 | 0.0087 | 2.47E-11 |
| MTNR1B | rs6483213 | 11 | 92725321 | a | 0.4931 | -0.0214 | 0.0019 | 1.07E-28 |
| MTNR1B | rs271046 | 11 | 92727874 | t | 0.3414 | 0.0197 | 0.0032 | 4.48E-10 |
| C2CD4A | rs4502156 | 15 | 62383155 | t | 0.4215 | 0.0116 | 0.002 | 3.12E-09 |
| C2CD4A | rs7163757 | 15 | 62391608 | t | 0.4755 | -0.0112 | 0.002 | 8.88E-09 |
| C2CD4A | rs8037894 | 15 | 62394264 | c | 0.4915 | -0.011 | 0.002 | 1.94E-08 |
| C2CD4A | rs6494307 | 15 | 62394690 | c | 0.4155 | 0.012 | 0.002 | 8.68E-10 |
| C2CD4A | rs7167878 | 15 | 62396189 | a | 0.4744 | -0.0113 | 0.0019 | 5.17E-09 |
| C2CD4A | rs7172432 | 15 | 62396389 | a | 0.4206 | 0.011 | 0.002 | 1.73E-08 |
| C2CD4A | rs7173964 | 15 | 62396942 | a | 0.3816 | -0.0113 | 0.002 | 7.44E-09 |
| FN3KRP | rs1046875 | 17 | 80685426 | a | 0.317 | 0.0123 | 0.0021 | 3.87E-09 |
| FN3KRP | rs1046896 | 17 | 80685533 | t | 0.3178 | 0.0142 | 0.002 | 6.64E-13 |
| ZNF750 | rs9906115 | 17 | 80791469 | a | 0.2649 | 0.013 | 0.0022 | 2.21E-09 |
| ZNF750 | rs7225515 | 17 | 80795783 | a | 0.2483 | 0.0122 | 0.0022 | 2.42E-08 |
| TBCD | rs4075209 | 17 | 80800027 | t | 0.2473 | 0.0123 | 0.0022 | 1.98E-08 |
| TBCD | rs9897951 | 17 | 80810662 | t | 0.2179 | -0.0123 | 0.0022 | 2.27E-08 |
